# N1-Methyladenosine-Related lncRNAs Are Potential Biomarkers for Predicting Prognosis and Immune Response in Uterine Corpus Endometrial Carcinoma

**DOI:** 10.1155/2022/2754836

**Published:** 2022-07-31

**Authors:** Jinhui Liu, Rui Geng, Zihang Zhong, Yixin Zhang, Senmiao Ni, Wen Liu, Mulong Du, Jianling Bai

**Affiliations:** ^1^Department of Gynecology, The First Affiliated Hospital of Nanjing Medical University, Nanjing, 210029 Jiangsu, China; ^2^Department of Biostatistics, School of Public Heath, Nanjing Medical University, 101 Longmian Avenue, Jiangning District, Nanjing 211166, China

## Abstract

Uterine corpus endometrial carcinoma (UCEC) is a malignant disease that, at present, has no well-characterised prognostic biomarker. In this study, two clusters were identified based on 28 N1-methyladenosine- (m1A-) related long noncoding RNAs (lncRNAs), of which cluster 1 was related to immune pathways according to the results of an enrichment analysis. We further observed better prognosis in patients with higher levels of immune cell infiltration, tumor mutation burden, microsatellite instability, and immune checkpoint gene expression. In addition, through Cox regression analysis and least absolute shrinkage and selection operator regression analysis, 10 m1A-related lncRNAs (mRLs) were employed to build a prognosis model. We found that people in higher risk categories had a poorer survival probability than those in lower risk. Low-risk samples were enriched with immune-related pathways, while the high-risk group was similar to the definition of the “immune desert” phenotype, which was associated with decreased immune infiltration, T cell failure, and decreased tumor mutation burden, while also being insensitive to immunotherapy and chemotherapy. This mRL-based model has the ability to accurately predict the prognosis of UCEC patients, and the mRLs could become promising therapeutic targets in enhancing the response of immunotherapy.

## 1. Introduction

The morbidity and mortality of UCEC are continually rising, as there were 417367 new cases and 97370 deaths in 2020 [1]. The prognosis of most patients is good in early stages [2]; however, once metastasis occurs, the survival rate is significantly reduced [3]. Traditional prediction methods for assessing the progress and prognosis of UCEC have proven to be inaccurate [4]; therefore, establishing a prediction model to provide a new prognostic signature for UCEC is required urgently. “Cold tumor” is an immune failure type associated with T cell failure and poor immunotherapeutic effect [5]. Effectively identifying a hot tumor and altering cold tumor to hot tumor will improve the effect of immunotherapy [6].

RNA methylation involves widely taking part in RNA post transcriptional modification, and its imbalance is associated with the genesis of malignant cancers [7]. m1A is a new RNA post transcriptional modification [8]. We know little about its impact on tumor development and its biologic mechanisms, although a previous study has observed the imbalance of m1A-related enzymes in gastrointestinal tumor samples [9]. lncRNAs are a new encoding RNA, which are widely involved in the development of cancer, cardiovascular disease, and diabetes [10]. lncRNAs can take part in tumor growth and metastasis through transcriptional and post transcriptional mechanisms [11]. Recently, it has been found that lncRNAs may feature an abnormal expression in UCEC and many other cancers [12, 13].

In this study, we identified 10 m1A-related lncRNAs (mRLs) that can be used as prognostic signatures of UCEC, verifying that this model can judge the prognosis of UCEC and offer a rationale for their therapy.

## 2. Materials and Methods

### 2.1. Data Acquisition

RNA sequencing transcription data, somatic mutation (VarScan version), and clinical data of UCEC patients were gathered from The Cancer Genome Atlas (TCGA) database (https://portal.gdc.cancer.gov/) [14]. Samples with missing overall survival (OS) values and no clinical follow-up information were excluded. A total of 511 UCEC samples and corresponding clinical profiles, such as age, stage, grade, and histological type, were applied for further study (Table S1). All the samples were separated into a training and testing set by the 1 : 1 ratio randomly through the “Caret” package. The information of clinicopathological features is shown in Table S1.

### 2.2. Identification of m1A-Related Genes (mRGs) and mRLs

Ten mRGs (*TRMT10C*, *TRMT61B*, *TRMT6*, *TRMT61A*, *ALKBH1*, *ALKBH3*, *YTHDF1*, *YTHDF2*, *YTHDF3*, *YTHDC1*) were obtained from previous studies [9]. The expression data of mRGs and mRLs was gathered from the TCGA database. In light of the Pearson analysis, we identified 621 mRLs which correlated with mRGs. The inclusion criteria was |*r*| >0.4 and *P* < 0.001. Then, univariate regression analysis was used to find mRLs with a potential prognostic value of UCEC (Table S2). The heat map showed the expression of 28 mRLs, and the box plot showed the distinctions in their expression between tumors and normal samples.

### 2.3. Unsupervised Consensus Clustering Analysis

Through the “ConsensusClusterPlus,” package we constructed a consistency cluster consisting of 28 mRLs [15]. The clustering divided the samples into multiple different groups according to the provided characteristics. The number of possible clusters (*k*) was defined in the range of 2 to 9 in order to avoid excessive numbers of groupings that would not be clinically useful.

### 2.4. Establishment of the mRL-Based Model (MRLM)

The entire TCGA set was randomly assigned into two subtypes, and the MRLM was constructed in light of the training group, with the entire set and testing set being employed to test the MRLM. The R package “glmnet” was employed to conduct least absolute shrinkage and selection operator (LASSO) regression, which showed that the expression level of 10 mRLs had correlation with OS in UCEC patients. A prognostic model in light of the 10 mRLs was then established. The risk score was achieved in line with the formula as follows: risk score = *β*_1_ × lncRNA_1_ + *β*_2_ × lncRNA_2_ + ⋯+*β*_*n*_ × lncRNA_*n*_, where *β*_*n*_ represents the coefficient of lncRNAs related to survival.

### 2.5. Quantitative Real-Time Reverse Transcription-Polymerase Chain Reaction (qRT-PCR)

lncRNAs from UCEC and normal tissues were extracted using the TRIzol reagent (Invitrogen). Before, with regard to reverse transcription to cDNA, a 4x GDNA wiper mix (vazymer323-01) was applied to remove residual genomic DNA from the total lncRNA. Complementary lncRNA was synthesised through a PrimeScript RT reagent kit. Real-time quantification was conducted through the SYBR Premix Ex Taq Kit (TaKaRa DRR041). The relative expression level of the target gene was standardised by the GAPDH and 2^-△△Ct^ method. The qRT-PCR primers are listed in Table S3.

### 2.6. Analysis of Protein Expression

We used the Pearson correlation analysis method to find m1A RNA corresponding to the 10 mRLs before downloading the expression files of m1A methylated proteins from UALCAN (http://ualcan.path.uab.edu). UALCAN is an open platform that contains cancer genomics, transcriptomics, and proteomics data [16, 17].

### 2.7. Evaluation of Predictive Ability of the MLRM

In the light of the median value of the risk score, the samples were separated into two subgroups: a high-risk group and a low-risk group [18]. Kaplan-Meier was used to evaluate OS [19, 20]. The principal component analysis (PCA) showed the ability of our model to distinguish patients into different UCEC subtypes [21].The distribution of clinical characteristics between the subtypes was displayed by the “pheatmap” R package. Univariate and multivariate Cox regression analyses were employed to evaluate whether the risk score was an independent predict factor of UCEC. Other models were built based on a multivariate Cox regression analysis, while ours was constructed through a LASSO regression analysis. In order to make them comparable, we employed a multivariate Cox regression analysis to calculate the risk score of each sample. The corresponding genes were incorporated into the four models, and the ROC curve was then drawn. The samples were divided into a high-risk group and a low-risk group according to the median risk score.

### 2.8. Constructing and Verifying a Predictive Nomogram

In light of the risk score and clinicopathological characteristics, a nomogram was established. A correction curve, calculated through a Hosmer-Lemeshow test, verified that the actual results were consistent with the predicted results [22]. The area under the curve (AUC) and the receiver operating characteristic (ROC) curve were employed to calculate the diagnosis and prognosis value of clinical characteristics [23].

### 2.9. Enrichment Analysis

Gene set enrichment analysis (GSEA) was employed to conduct an enrichment analysis of two clusters and two risk groups. By aggregating gene expression changes into gene sets, users can achieve enrichment scores which allow them to deeply understand how biological pathways and processes have been influenced [24]. Here, we showed the first five terms of the Kyoto Encyclopedia of Genes and Genomes (KEGG) analysis.

### 2.10. Analysis of Immune Cell Features

CIBERSORT can quantitatively evaluate immune cell components from complex gene expression data in tissues [25]. Via CIBERSORT (http://cibersort.stanford.edu/), our study analysed the composition and infiltration level of 21 invasive immune cells.

### 2.11. Assessment of Tumor Microenvironment (TME)

“ESTIMATE” is a new algorithm that utilises the features of the tumor tissue transcription spectrum to infer stromal cells and immune cells in malignant tumor tissues [26]. This exploration was conducted using the R package “estimate,” and the score reflected the ratio of immune and stromal components in TME [27]. Single-sample gene set enrichment analysis (ssGSEA) was used to verify the differences of immune cells and immune function between the groups. ssGSEA worked at the single sample level and was an extension of the GSEA method [28].

### 2.12. Comparison of Cancer Stem Cells (CSCs)

CSCs have the potential for self-renewal and differentiation, making them crucial in the occurrence and treatment of cancers. The mRNA expression-based stemness index (mRNAsi) describes the similarity between tumors and stem cells, which is a quantitative form of CSC. UCEC samples achieved from TCGA were used for comprehensive analysis to obtain the differences in mRNAsi among groups [29].

### 2.13. Prediction of Immunotherapy Response

Induced pluripotent stem cells (IPS), ranging from 0 to 10, were evaluated in the light of the gene expression *Z* score, with higher IPS representing higher immunogenicity [30]. The results were downloaded from The Cancer Imaging Archive (TCIA) database [31]. Tumor mutation burden (TMB) indicates the number of somatic mutations in the genome sequence, which can be employed to screen patients who have a higher probability of response to immune checkpoint inhibitors (ICIs) [32]. Microsatellite instability (MSI) refers to the change of allele size between tumor tissue and its corresponding normal tissue. Profiles of MSI were also downloaded from the TCIA database, while genomic mutation data was gathered from the TCGA database. We used GISTIC_2.0 to identify significant amplification and deletion on chromosomes and to obtain the GISTIC score. The burden of copy number variation (CNV) gain or loss was evaluated by GenePattern (https://cloud.genepattern.org) [33].

### 2.14. Analysis of Drug Sensitivity

The R package “PRROPHIC” was applied to evaluate the half maximum inhibitory concentration (IC50) of four chemotherapeutics in two risk groups [34]. The connectivity map is an important database in the field of pharmacogenomics which is aimed at finding the functional relationship between drugs, genes, and diseases through gene expression [35]. In order to explore the mechanism of action (MoA) and drug targets in more detail, we used a connectivity map (CMap) to conduct further analysis (https://clue.io/) [36].

### 2.15. Statistical Analysis

Statistical tests were conducted in light of R version 4.1.0. Statistical significance was defined as *P* < 0.05. The distinction between the two subgroups was calculated by a Student *t*-test and variance analysis. A Kaplan-Meier analysis was applied to compare the OS differences between the groups.

## 3. Results

### 3.1. Identification of Prognostic mRLs

Figure S1 shows the flow chart of our study. The expressed information of 10 mRGs and lncRNAs were downloaded from the TCGA database. lncRNAs closely related to at least one of the 10 mRGs were considered to be mRLs, with 621 mRLs being identified (Figure S1B shows the network of 10 mRGs and 621 mRLs). Finally, the Sankey diagram in Figure S1C visualises the m1A-lncRNA coexpression network. Figure S1D performs the correlation between m1A genes and mRLs in TCGA.

**Figure 1:**
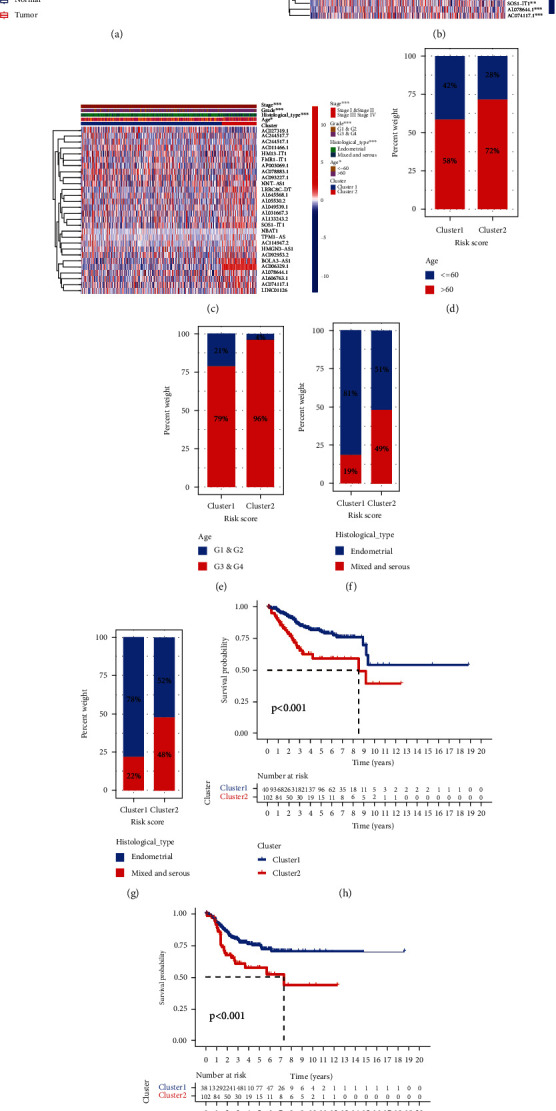
Different clinicopathological features and survival probability of the two UCEC subtypes. (a) Box plot of 28 mRL expressions in normal and tumor tissues. (b) Heat map of 28 mRL expressions in normal and tumor tissues. (c) The different expressions of mRLs and their clinicopathological features between the two clusters are shown by heat map. The proportions of age (d), grade (e), histological type (f), and stage (g) between the two clusters were compared. (h) The OS rate of UCEC patients in the two groups was calculated by the Kaplan-Meier curve. (i) Kaplan-Meier curves of disease-free survival for patients with UCEC in two clusters (cluster 1/2).

Combined with clinical information, 511 samples were obtained and distributed to training and testing sets. Through univariate regression analysis on the training set, we identified 28 mRLs with a potential prognostic value of UCEC (BOLA3-AS1, AC078883.1, AC093227.1, AC027319.1, AL078644.1, AL049539.1, HM13-IT1, AL645568.1, NBAT1, FMR1-IT1, LRRC8C-DT, AL133243.2, HMGN3-AS1, TPM1-AS, AC006329.1, AP003096.1, NNT-AS1, AC074117.1, SOS1-IT1, LINC01126, AL606763.1, AC092953.2, AL031667.3, AL035530.2, AC114947.2, AC244517.7, AC244517.1, and AC011466.1) (Figure S1B). The expression levels of these 28 mRLs were different between the UCEC and normal tissues (Figures 1(a) and 1(b)).

### 3.2. Consistency Clustering Analysis

According to the similarity ratio and fuzzy clustering measure, we identified that the clustering stability will be best when *k* = 2. Figure S2A-S2B visualise the change of consistency clustering and cumulative distribution function (CDF) of AUC from *k* = 2 to *k* = 9. Consistency cluster and tracking plot also performed that when *k* = 2, the clustering result is satisfactory (Figure S2C-S2D). In light of *k* = 2, 511 samples were separated into two subtypes: cluster 1 (*n* = 409) and cluster 2 (*n* = 102).

### 3.3. Clinical Characteristics and Prognosis of the Two Subtypes

The heat map showed the expressed level of 28 mRLs and the distribution of clinical characteristics in the two subtypes (Figure 1(c)). Patients in cluster 2 are older and at a more advanced stage, grade, and histological type. In cluster 1, age ≤ 60 accounted for 42% and age > 60 accounted for 58%, grade 1 and grade 2 accounted for 21% and grade 3 and grade 4 accounted for 79%, patients belonging to the essential histological type accounted for 81% and 19% belonged to the mixed and serous type, and stage I and stage II accounted for 78% and stage III and stage IV accounted for 22%. Alternatively, in cluster 2, age ≤ 60 accounted for 28% and age > 60 accounted for 72%; grade 1 and grade 2 accounted for 4% and grade 3 and grade 4 accounted for 96%; patients belonging to the endogenous histological type accounted for 51%, while patients belonging to the mixed and serial type accounted for 49%; and stage I and stage II accounted for 52% and stage III and stage IV accounted for 48% (Figures 1(d)–1(g)). At the same time, patients in cluster 2 had lower OS and disease-free survival (DFS) than those in cluster 1 (*P* < 0.001) (Figures 1(h) and 1(i)).

### 3.4. Cluster 1 Had Higher Infiltration Level of Immune Cells

**Figure 2:**
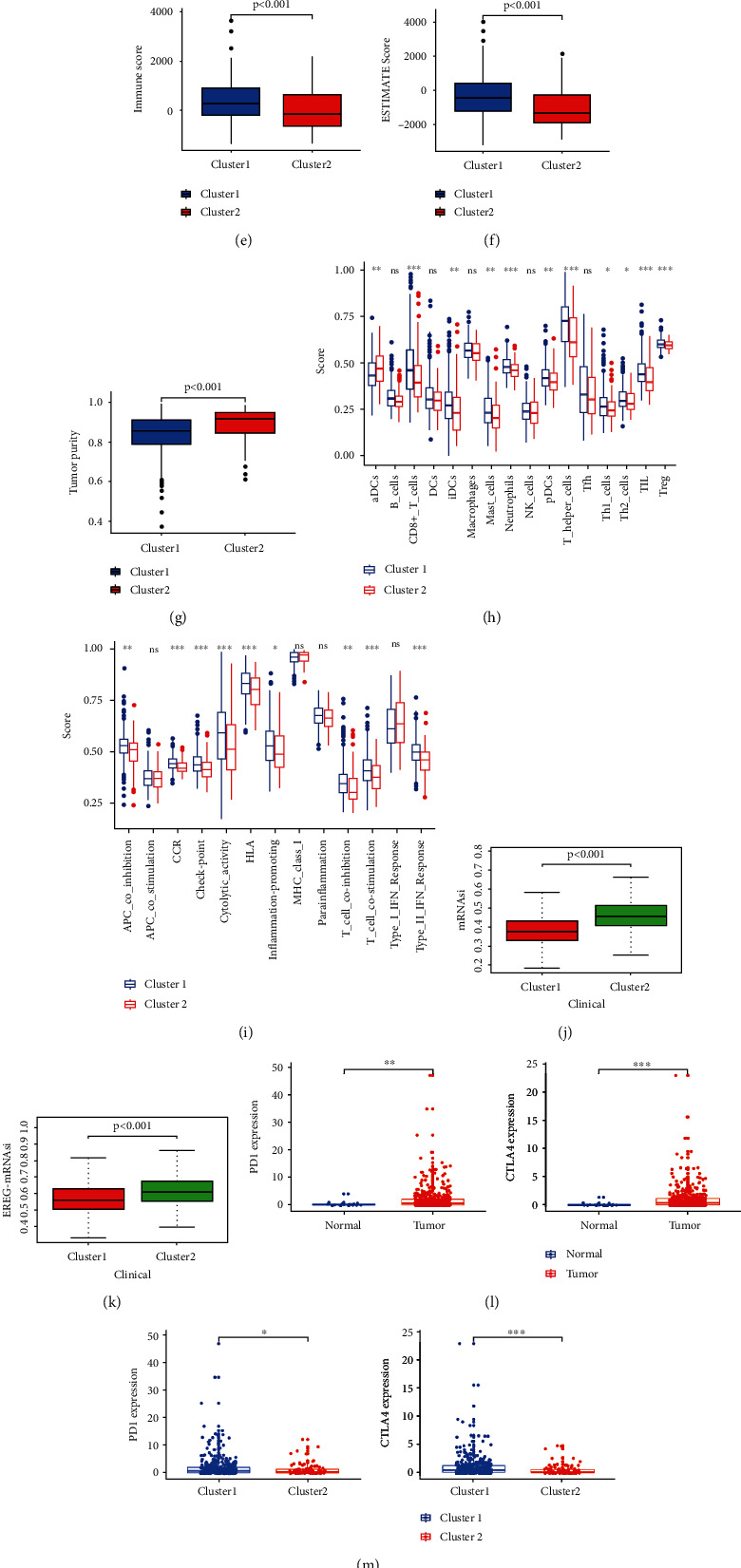
Immunoassay of two clusters. (a) Cluster 1 was enriched with immune-related pathways. (b) Cluster 2 was enriched with some tumor-related pathway. (c) The infiltrating levels of 22 immune cell types in cluster 1/2 subtypes in the TCGA cohort. ^∗^*P* < 0.05, ^∗∗^*P* < 0.01, and ^∗∗∗^*P* < 0.001. Immune score (d), stromal score (e), ESTIMATE score (f), tumor purity (g), immune cells (h), immune function (i), mRNAsi (j), and EREG-mRNAsi (k) in two clusters. (l) The difference of the expression of PD1 and CTLA4 between normal and tumor tissues. (m) The difference of the expression of PD1 and CTLA4 between the two clusters. (n) The correlation between PD1 and mRLs. (o) The correlation between CTLA4 and mRL (p) IPS analysis.

Cluster 1 was enriched with a chemokine signaling pathway, complement and coagulation cascades, a cytokine, a cytokine receptor interaction, and an intestinal immune network for IGA production and natural killer cell-mediated cytotoxicity (Figure 2(a)). Cluster 2 was enriched with a cell cycle, DNA replication, homologous recombination, mismatch repair, and P53 signaling pathway (Figure 2(b)). We then evaluated the fraction of 21 tumor immune infiltration cells in the two clusters (Figure 2(c)). Regulatory T cells (Tregs) and neutrophils had a higher fraction in cluster 1 than in cluster 2, while memory-activated CD4 T cells, follicular helper T cells, gamma delta T cells, and M1 macrophages had a higher fraction in cluster 2. Immune score, stromal score, and ESTIMATE score were all higher in cluster 1 (Figures 2(d)–2(f)), while the tumor purity of samples in cluster 1 was lower than that of samples in cluster 2 (Figure 2(g)). The score of immune cells and immune function in cluster 1 was significantly higher than that in cluster 2 (Figures 2(h) and 2(i)). Both mRNAsi and EREG-mRNAsi were higher in cluster 2, indicating that the degree of cell differentiation was low and the characteristic of stem cells was strong (Figures 2(j) and 2(k)). We then assessed the expressed level of immune checkpoints between different groups. The levels of PD1 and CLAT4 in tumors were significantly higher than those in normal tissues, and it was also higher in cluster 1 than in cluster 2 (Figures 2(l) and 2(m)). After that, we further analysed the association between PD1 and mRLs (Figure 2(n)). The association between CTLA4 and mRLs was also assessed (Figure 2(o)). In the TCGA cohort, the expressed level of PD1 was closely correlated with BOLA3–AS1, AC078883.1, FMR1–IT1, NNT–AS1, AC074117.1, LINC01126, AL606763.1, AC244517.7, and AC244517.1. The result achieved from IPS analysis showed that the score of IPS (*P* = 0.011), IPS_ctla4 (*P* = 0.022), IPS_pdl1_pd1_pdl2 (*P* = 0.019), and IPS_pdl1_pd1_pdl2_ctla4 (*P* = 0.002) was higher in cluster 1, which meant that cluster 1 had higher immunogenicity (Figure 2(p)).

### 3.5. Prediction of Immunotherapy Response

**Figure 3:**
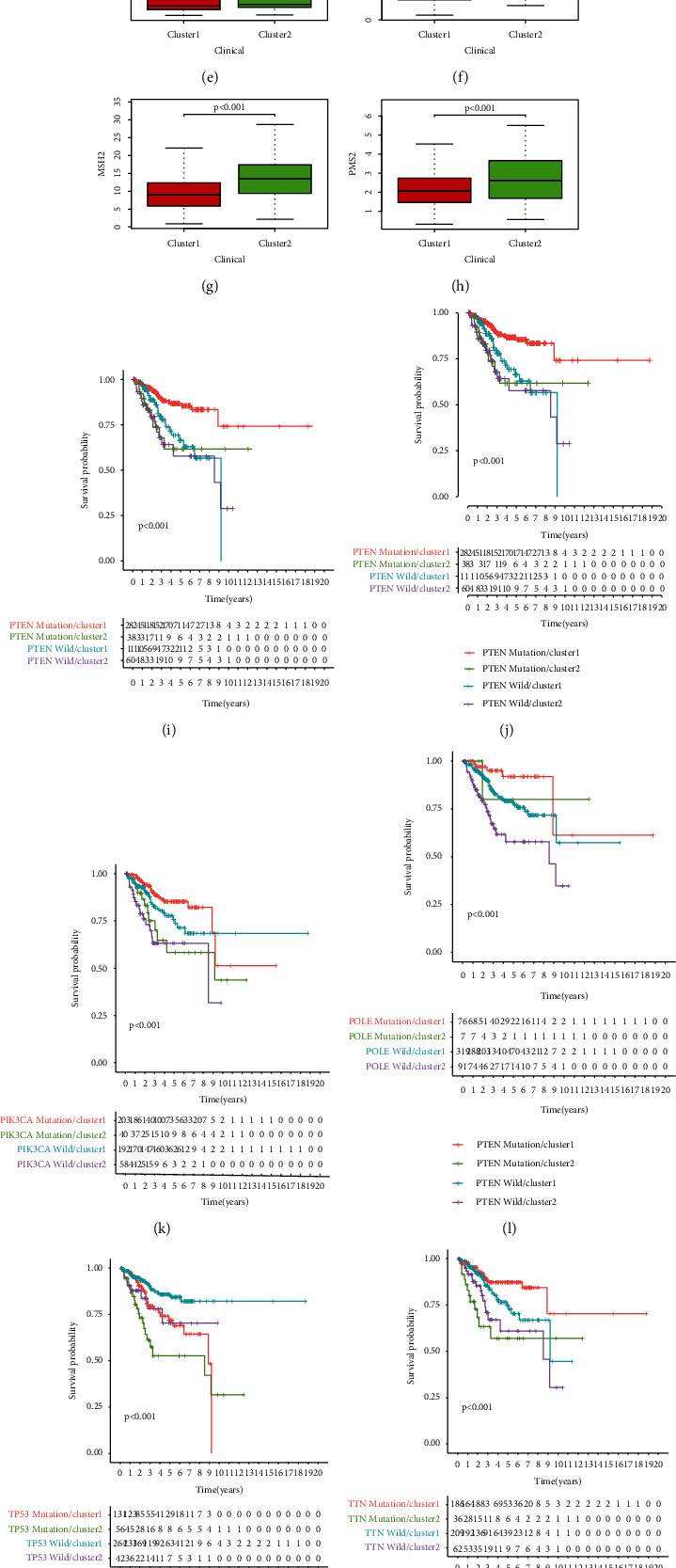
Prediction of immunotherapy response and drug sensitivity. (a) The different survival probability of patients with different TMB. (b) The survival probability of patients with different TMB in different clusters. The difference of TMB (c) and stochastic mutation nut (d) between the clusters. Comparison of MSI (e), MSH2 (f), MSH6 (g), and PMS2 (h) between the clusters. The influence of mutations of PTEN (i), ARID1A (j), PIK3CA (k), POLE (l), TP53 (m), and TTN (n) in the survival rate. The sensitivity of the two clusters to cisplatin (o), doxorubicin (p), etoposide (q), and paclitaxel (r) was different.

The survival probability of cluster 2 was lower than that of cluster 1 (*P* < 0.001) (Figure 3(a)), while the survival probability of patients with L-TMB in cluster 2 was lower than that of other patients (*P* < 0.001) (Figure 3(b)), although there was no statistical distinguishing factors in TMB and somatic mutation nut between the subtypes (Figures 3(c) and 3(d)). MSI, another index to judge the effect of immunotherapy, was lower in cluster 1 than in cluster 2 (*P* = 0.026) (Figure 3(e)). The expression levels of mismatch repair genes, MSH2, MSH6, and PMS2, which were involved in the occurrence of MSI, were higher in cluster 2 than in cluster 1 (*P* < 0.001) (Figures 3(f)–3(h)). The mutations of PTEN, ARID1A, PIK3CA, POLE, TP53, and TTN can influence the OS of UCEC patients, while the survivability of cluster 2 was lower (*P* < 0.001) (Figures 3(i)–3(n)).

### 3.6. Drug Sensitivity Analysis

Four common chemotherapeutic drugs, cisplatin, doxorubicin, etoposide, and paclitaxel, showed higher etoposide sensitivity in cluster 1, meaning that this cluster may be effective in the treatment of chemotherapeutic drugs (Figures 3(o)–3(r)).

### 3.7. Establishment and Validation of the MRLM

**Figure 4:**
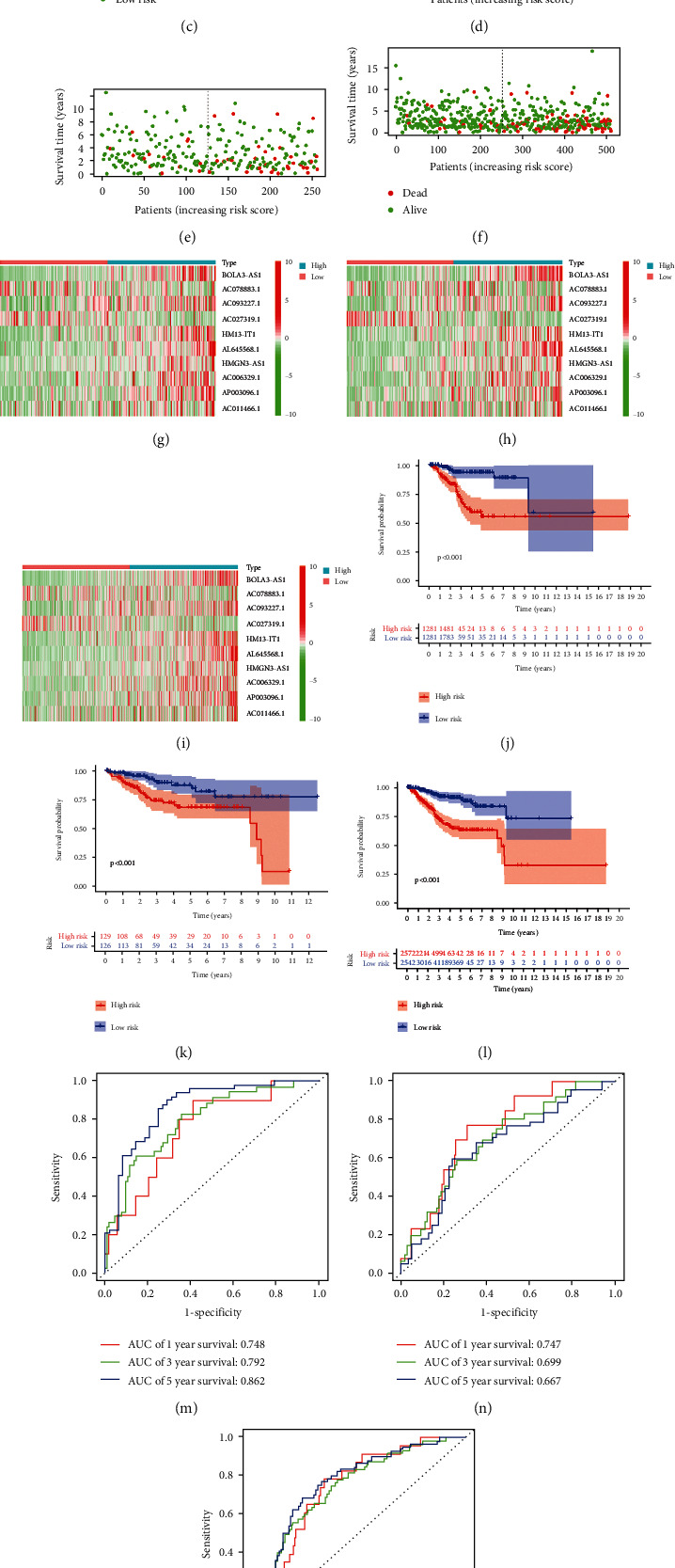
Predictive value of risk model constructed by mRL-indifferent patient sets. Training set (a), testing set (b), and entire set (c) were divided into high- and low-risk groups according to the median risk score. Distribution of survival time and survival status between high-risk group and low-risk group in training set (d), testing set (e), and entire set (f). The heat map of cluster analysis shows the expression levels of ten mRLs in the training set (g), testing set (h), and entire set (i). Kaplan-Meier survival curve of OS in the low-risk group and high-risk group in the training set (j), testing set (k), and entire set (l). Prediction sensitivity in the training set (m), testing set (n), and entire set (o) in 1-3 and 5 years.

A LASSO Cox analysis was performed on 28 mRLs screened through a univariate regression analysis (Figure S3A-3B), and 10 mRLs with independent prognostic values were identified to establish the model (Table S4). Figures 4(a)–4(c) show the distribution of risk level between different risk subtypes. The survival status and survival time of patients in the groups are indicated in Figures 4(d)–4(f). Figures 4(g)–4(i) show the relative expression criteria of 10 mRLs for every patient. After that, qRT-PCR was used to compare the difference of the mRL expression between normal and tumor tissue (Figure S4). The results showed that AC006329.1 and AC027319.1 had higher expression levels in tumor tissues, while the expressions of AC011466.1, AC093227.1, and BOLA3–AS1 were significantly higher in normal tissues. Pearson correlation analysis found that 10 mRGs (*TRMT10C*, *TRMT61B*, *TRMT6*, *TRMT61A*, *ALKBH1*, *ALKBH3*, *YTHDF1*, *YTHDF2*, *YTHDF3*, and *YTHDC1*) had a strong correlation with at least one lncRNA, which we used to build the model. Then, expression profiles of m1A methylated proteins were downloaded, with the results being shown in Figure S5. It was noted that the protein expression of most of these genes was different in normal tissues and UCEC tissues. The results obtained from PCA indicated that the risk score had greater discrimination for UCEC patients (Figure S6). Survival analysis identified that the OS of low-risk people was higher than that of high-risk patients (*P* < 0.001) (Figures 4(j)–4(l)). As shown in Figures 4(m)–4(o), the model had high prediction sensitivity, whether in the testing set or training set. In the two clusters, the expression of 10 mRLs was different, and the corresponding survival probability was also significantly different (*P* < 0.001).

### 3.8. The High-Risk Group Had More Advanced Symptoms and Poorer Prognoses

We calculated the differences in clinical characteristics between groups and plotted a heat map (Figure S7A). Most cases in cluster 2 were at high risk. Higher risk scores were correlated with older age, as well as a more advanced stage, grade, histological type, and lower immunity score (Figure S7B-S7C). The survival probabilities of different clinicopathological characteristics patients between the two groups were then compared (Figure S7D). With different clinical characteristics, the survival probability of the low-risk group was higher than that of the high-risk group (*P* < 0.05), except in patients with mixed and serous histological types. Lower expression in AC027319.1 and AC078883.1 corresponded to lower OS, while lower expression levels of BOLA3-AS1, HMGN3-AS1, HM13-IT1, AC006329.1, AC093227.1, and AL645568.1, AP003096.1 corresponded to higher OS (Figure S7E).

### 3.9. Construction and Verification of Nomogram

**Figure 5:**
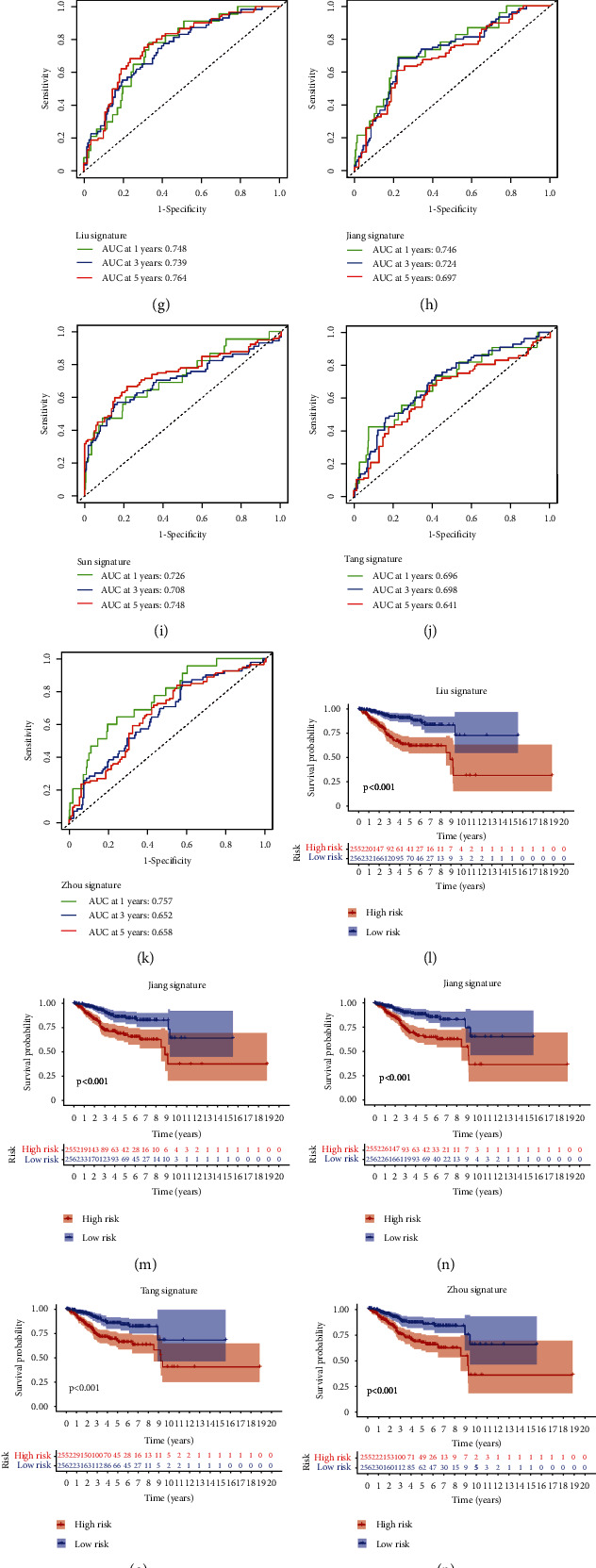
Construction and evaluation of a prognostic nomogram. The area under the ROC curve (AUC) of risk score and single clinical characteristics in 1 year (a), 3 years (b), and 5 years (c). The AUCs of risk score combine with clinical characteristics in 1 year (d), 3 years (e), and 5 years (f). ROC (g–k) and Kaplan-Meier curve (l–p) of our model and the other four published prediction models. (q) Restricted mean survival time curves for all five prognostic risk models. (r) The C-index of our model is higher than other clinical features. (s) The consistency index of 5 prognostic factors including risk score. (t–v) The calibration plot of the nomogram predicts the probability of the 1-, 3-, and 5-year OS.

A Cox regression analysis of the testing, training, and entire set showed that the risk score was an independent predicting factor of UCEC (Table 1). For the purpose of evaluating the prediction accuracy of MRLM, we compared the true positive rates predicted by MRLM, clinical factors, and the model in combination with the clinical factors, respectively. The ROC curves of 1, 3, and 5 years have been performed in Figures 5(a)–5(c). The AUCs of MRLM in the three different years were 0.656, 0.749, and 0.769, respectively, making them higher than the clinical characteristic as a whole. It is worth mentioning that the prediction effect of combining MRLM with clinical factors was better (Figures 5(d)–5(f)). In order to verify the performance of our model, we chose four other reported models to compare, namely, a 5-gene signature [37], a 13-gene signature [38], a 5-gene signature [39], and an 11-gene signature [40]. To make the models comparable, we assessed the risk score of each sample in all TCGA cohorts via a multivariable Cox regression analysis. We then included the corresponding genes in these four models and drew the ROC curves. In all, the AUC of these models was lower than that of MRLM (Figures 5(g)–5(k)). There were significant differences between the prognosis of the high- and low-risk groups of the models (Figures 5(l)–5(p)), and RMS could evaluate the prediction effect at different time points. Compared with other models, ours performed fairly well (Figure 5(q)). The limited mean survival software package was used to calculate the C-index of all prognostic features, and the C-index of our model was higher than that of other clinical features (Figure 5(r)). According to the above results, we inferred that the risk score evaluated by 10 mRLs had an accurate prognosis ability. Through comparison with clinical characteristics, the risk level of the MRLM performed an outstanding predicted value through a nomogram (Figure 5(s)). The correlation diagram showed that the observed OS ratios in 1, 3, and 5 years were consistent with the predicted ratios (Figures 5(t)–5(v)).

### 3.10. Estimation of TME and Response to ICI on the Base of the PRLs

**Figure 6:**
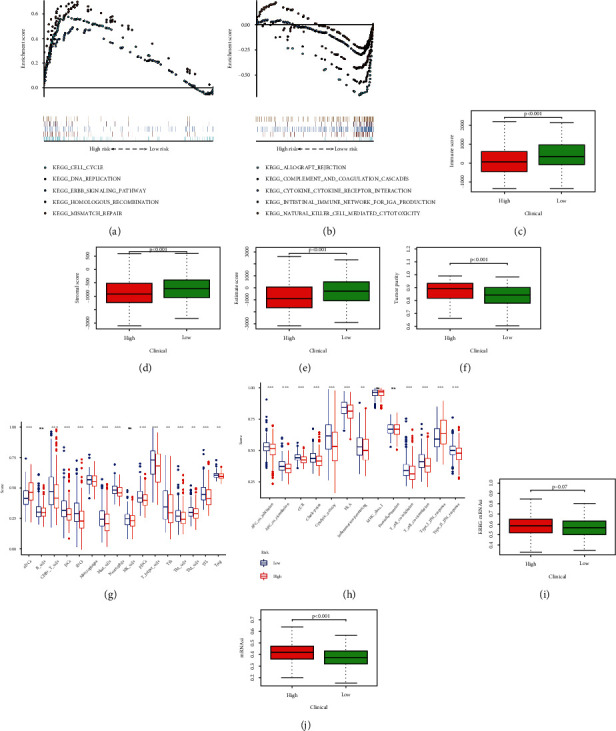
The results of ESTIMATE, ssGSEA, and drug sensitivity analysis. (a) High-risk group was enriched with some tumor-related pathways. (b) Low-risk group was enriched with immune-related pathways. The difference of immune score (c), stromal score (d), ESTIMATE score (e), and tumor purity (f) in high- and low-risk groups. The difference of immune cells (g) and immune functions (h) between the two risk groups. EREG-mRNAsi (i) and mRNAsi (j) expression in two clusters.

**Figure 7:**
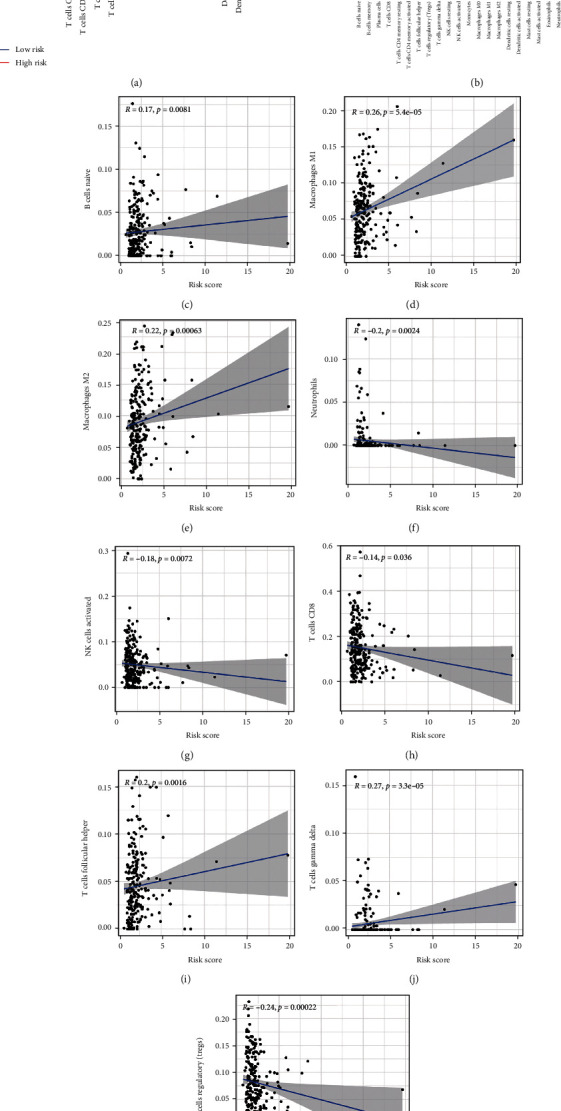
The correlation between tumor-infiltrating immune cells and the model. (a) Violin plot shows the difference of the fraction of each immune cells between the two risk groups. (b) The correlations between 21 tumor-infiltrating cells and mRLs. (c–k) The correlation between risk score and tumor-infiltrating immune cells.

**Figure 8:**
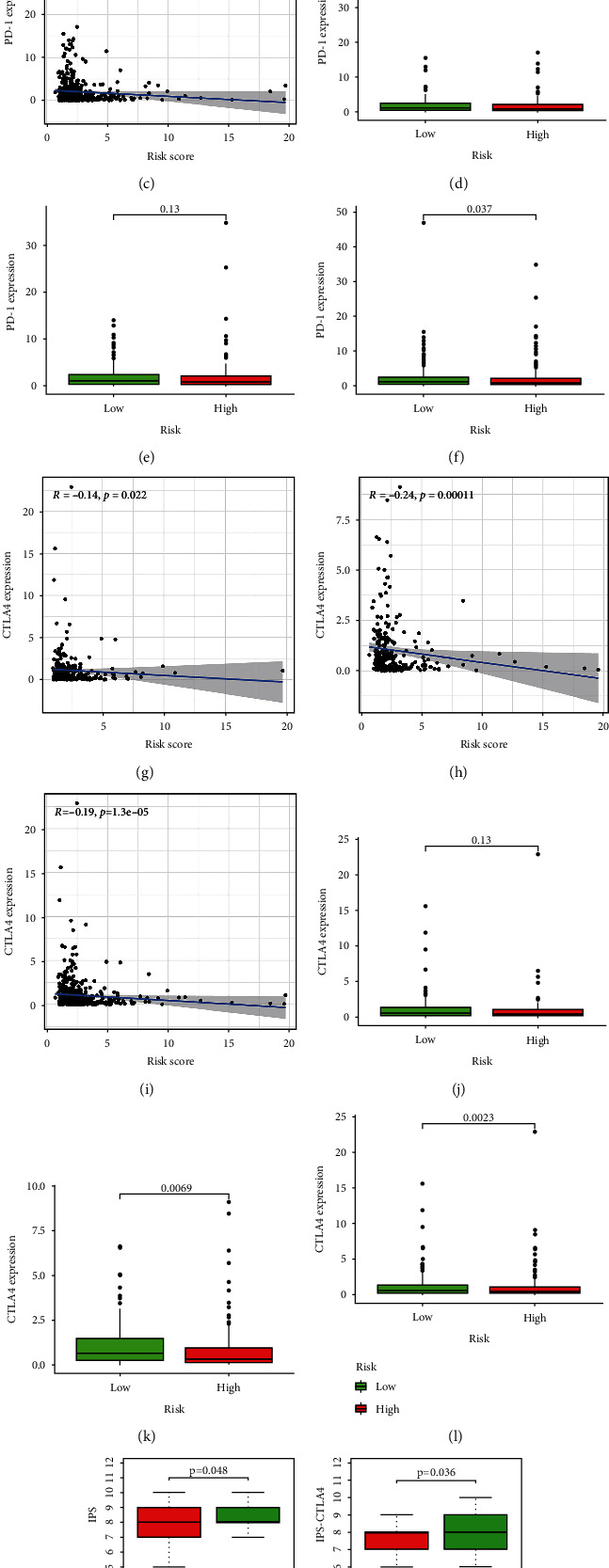
The correlations between immune checkpoint and risk score. The correlations between PD1 and risk score in the training set (a), testing set (b), and entire set (c). Different expression of PD1 between two risk groups in the training set (d), testing set (e), and entire set (f). The correlations between CTLA4 and risk score in the training set (g), testing set (h), and entire set (i). Different expression of CTLA4 between two risk groups in the training set (j), testing set (k), and entire set (l). The differences of IPS (m), IPS_ctla4 (n), IPS_ctla4_pdl1_pd1_pdl2 (o), and IPS_pdl1_pd1_pdL2 (p) in patients with different risks.

Through GSEA, it was found that the high-risk group was enriched in some tumor-related pathways (Figure 6(a)), while the low-risk group was mainly enriched in some immune-related pathways (Figure 6(b)). Immune cells and stromal cells are two crucial compositions of TME. We assessed the immune cell (Figure 6(c)) and stromal cell (Figure 6(d)) score in the two groups and added them to obtain an ESTIMATE score (Figure 6(e)). The score of patients at low risk was higher than that of patients at high risk (*P* < 0.01). A higher ESTIMATE score indicated lower tumor purity, which was consistent with our results (Figure 6(f)). There was also a distinction in immune cells and immune function between the groups. The score of immune cell and immune function in high-risk groups was usually lower than that in low-risk groups (Figures 6(g) and 6(h)). Both mRNAsi (*P* < 0.001) and EREG-mRNAsi (*P* = 0.07) were higher in patients with a high-risk score, indicating that the degree of cell differentiation was low and the characteristics of stem cells was strong (Figures 6(i) and 6(j)). We then compared the relative percentage of 21 tumor immune infiltrating cells in the two risk groups (Figure 7(a)). There was a statistical distinction in the expression of multiple immune infiltrating cells between the two groups. Naive B cells (*P* = 0.005), follicular helper T cells (*P* = 0.003), M1 macrophages (*P* < 0.001), and M2 macrophages (*P* = 0.006) had higher expression in the high risk group, while CD8 T cells (*P* = 0.005), regulatory T cells (Tregs) (*P* = 0.001), gamma delta T cells (*P* < 0.001) and activated NK cells (*P* = 0.004) had higher expression levels in patients with low-risk scores. The expression of these immune infiltrating cells was closely related to mRLs (Figure 7(b)). After that, we assessed the relationships between the MRLM risk score and immune infiltrating cells. The risk score had positive relationships with naive B cells (*R* = 0.17, *P* = 0.0081), M1 macrophages (*R* = 0.26, *P* < 0.001), M2 macrophages (*R* = 0.22, *P* = 0.00063), follicular helper T cells (*R* = 0.2, *P* = 0.0016), and gamma delta T cells (*R* = 0.27, *P* < 0.001), while it had negative relationships with neutrophils (*R* = −0.2, *P* = 0.0024), activated NK cells (*R* = −0.18, *P* = 0.0072), CD8 T cells (*R* = −0.14, *P* = 0.036), and regulatory T cells (Tregs) (*R* = −0.24, *P* = 0.00022), which showed that the level of T cell infiltration was lower in the high-risk group (Figures 7(c)–7(k)). The results suggested that the score of MRLM could identify different features of immune cells. We then calculated the relationships between the immune checkpoint genes PDCD1 and CTLA4 and risk score (Figures 8(a)–8(l)). In the three sets, PDCD1 and CTLA4 were negatively related to the risk. There were expression differences between the risk groups, except in the training set. The expression level of immune checkpoints in patients with high-risk scores was low, and there may exist T cell failure. IPS analysis showed that scores of IPS, IPS-CTLA4, IPS-PD1-PD-L1-PD-L2, and IPS-PD1-PD-L1-PD-L2-CTLA4 (*P* < 0.05) were higher in patients at low risk, which also meant that low-risk patients were associated with higher immunogenicity (Figures 8(m)–8(p)). In light of the above results, we inferred that the high-risk UCEC belonged to cold tumor and may have had a poor response to immunotherapy.

### 3.11. Prediction of Immunotherapy Effect

**Figure 9:**
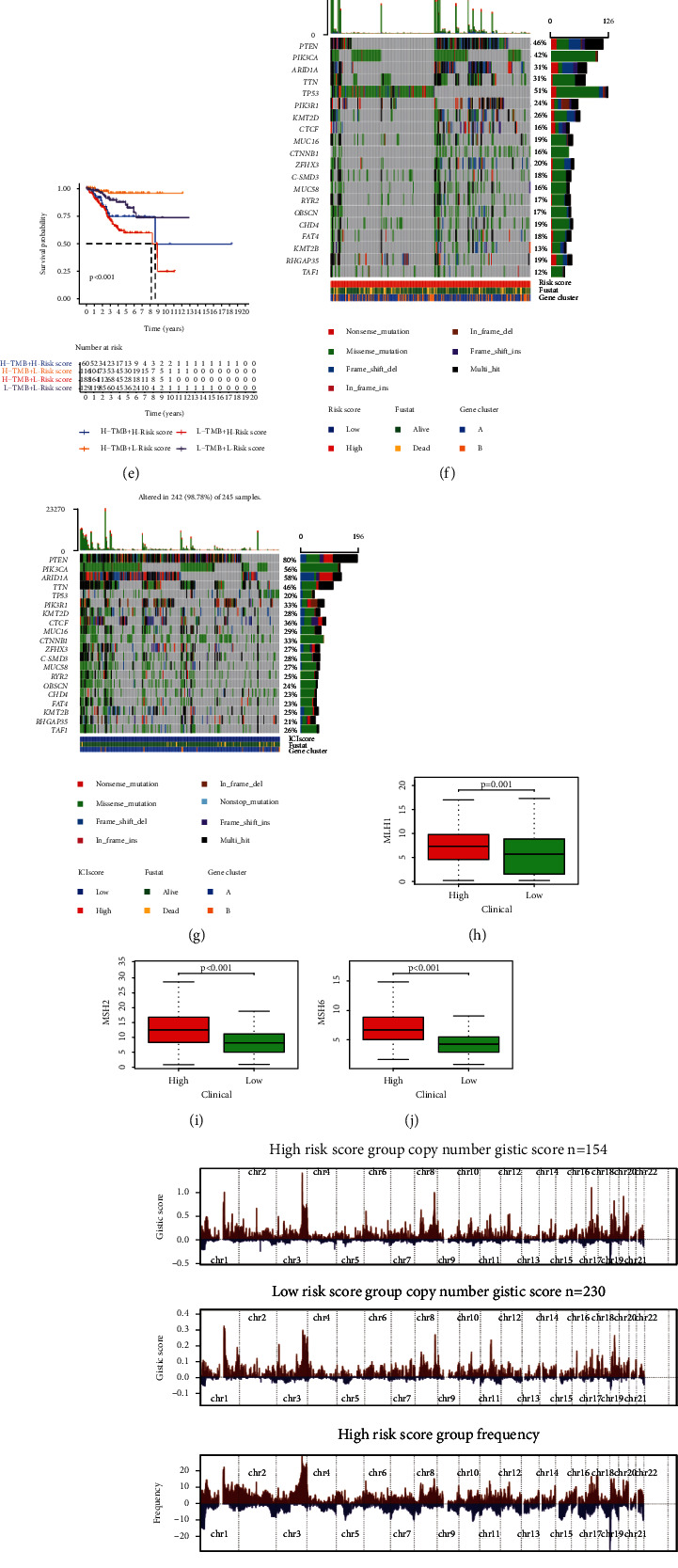
The relationship between immunotherapy response and survival. (a) The distribution of risk score and survival status of the two clusters. (b) The differences of TMB between the two groups. (c) There were significant differences in TMB between the two groups. (d) The survival probability of patients with different TMB. (e) The survival probability of patients with different TMB and risk scores. The mutation information of genes with high mutation frequency in the high- (f) and low-risk groups (g). Comparison of MSI (h), MSH2 (i), MSH6 (j), and PMS2 (k) between the groups. (l) Copy number profiles for the low- or high-risk groups, with gains in red and losses in blue. Gene segments are placed according to their location on chromosomes, ranging from chromosome 1 to chromosome 22. (m) Detailed cytoband with focal amplification (left) and focal deletion (right) in the low-risk score group generated with GISTIC_2.0 software. The *q* value of each locus is plotted horizontally. (n) Detailed cytoband with focal amplification (left) and focal deletion (right) in the high-risk score group generated with GISTIC_2.0 software.

Figure 9(a) shows the distribution of risk score and survival status of the two clusters. There were significant differences in TMB between different risk groups (*P* = 0.00044), and it was higher in low-risk patients (Figure 9(b)). TMB was negatively correlated with the risk (Figure 9(c)). The OS of the L-TMB group was low (Figure 9(d)). Meanwhile, under different TMB, the high-risk group had a significantly lower survival probability than the low-risk group (Figure 9(e)). The waterfall plot indicated the mutation information of genes with high mutation frequency in the high- (Figure 9(f)) and low-risk groups (Figure 9(g)). Microsatellite instability (MSI) was another tumor immune marker reflecting on the effect of immunotherapy. The expression levels of mismatch repair genes MLH1, MSH2, MSH6, and PMS2 were lower in the low-risk group (*P* < 0.001) (Figures 9(h)–9(k)).  Figure 9(l) shows the distribution of GISTIC scores calculated in light of the frequency and amplitude of gain and loss on all chromosomes in the high- and low-risk groups. Focal amplification and deletion of different chromosome regions was detected in both groups (Figures 9(m) and 9(n)). These results showed that the low-risk group had relatively high immunogenicity, while the high-risk group had relatively low immunogenicity. This meant that the stability of MSI in patients with a low-risk score was worse and the immunotherapy was effective. The somatic mutation count was higher in the low-risk group, whether in the training set, testing set, or entire set (Figure S8A-S8C). Among the three sets, PTEN, ARID1A, PIK3CA, POLE, and TTN had a higher mutation proportion of patients with a low-risk score, while TP53 was the opposite (Figure S8D-S8U).

### 3.12. Chemotherapy Drugs May Be More Effective in Low-Risk Patients

**Figure 10:**
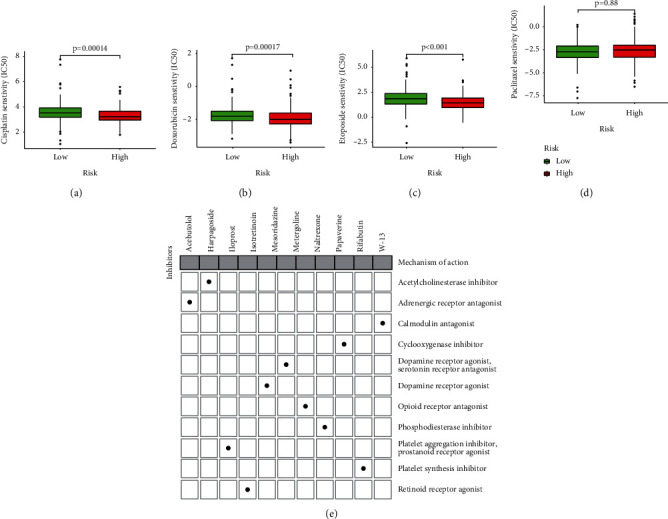
Drug sensitivity in high-risk score patients and the immune and molecular subtypes. The differences of sensitivity of patients to cisplatin (a), doxorubicin (b), etoposide (c), and paclitaxel (d) in high- and low-risk groups. (e) The heat map shows 11 action mechanisms obtained from the mode of action of the top 20 most closely related compounds in CMap.

Sensitivity analysis of four common chemotherapeutic drugs showed that cisplatin (*P* = 0.00014), doxorubicin (*P* = 0.00017), and etoposide (*P* < 0.001) had higher sensitivity in people with a low-risk score (Figures 10(a)–10(d)). This meant that the effect of chemotherapy drugs may cause better therapeutic efficacy in the low-risk group. CMap identified potential compounds on the basis of differentially expressed genes. Table S5 shows the top 20 drugs that were identified. Through MoA analysis of 20 compounds, it was found that the above compounds had 11 action mechanisms (Figure 10(e)).

## 4. Discussion

Although the study of UCEC treatment has made some progress [41], the mortality continues to rise, seriously threatening women's health. Many markers that can predict the prognosis of UCEC have been found [42]; however, so far, there is no unified index. lncRNAs have the potential to become immunotherapeutic targets and biomarkers for a UCEC prognosis due to the characteristics of its high efficiency, high tissue specificity, and high stability. More and more evidence indicates that the uncommon expression of lncRNAs may be associated with the occurrence and progression of a variety of kinks in tumors [43–48]. m1A is a common form of RNA modification, and it has a crucial effect on a variety of diseases, particularly in cancer [49]. It has been observed that there were disorders of m1A-related enzymes and a variety of genetic alterations in tumor samples [9], and so it could be applied as a prognostic signature for gastrointestinal cancer and pancreatic cancer [50]. At the same time, m1A has an important effect on maintaining the structure and function of noncoding RNAs (ncRNAs) [51].

In order to explore whether m1A can be employed as a sensitive molecular prognostic and diagnostic signature of UCEC, the prognostic characteristics based on mRLs were constructed and verified in this study.

Firstly, 10 mRGS were obtained from published articles, all of which were related to RNA metabolism [9]. The role of 10 mRGS in UCEC has not been reported, but they were related to the occurrence and development of some other tumors to varying degrees [52–61]. We identified 28 mRLs with a potential prognostic value of UCEC based on 10 mRGS combined with clinical data and statistical analysis. There were significant expression differences between normal tissues and UCEC patients which had the potential to distinguish the prognosis of UCEC patients.

Among the two subtypes divided by consistent cluster analysis, the clinicopathological characteristics of patients in cluster 2 were more serious: older, more advanced stages, more serious histological types, and worse survival probabilities. The outcomes of GSEA indicated that cluster 1 was enriched with immune-related pathways and cluster 2 was enriched with some tumor-related pathways. Immune desert tumors usually have poor immune infiltration and lack preexisting antitumor immunity [62]. The fraction of Tregs was lower in cluster 2, while the fraction of macrophase M1 was higher than in cluster 1, in line with the definition of the immune desert. Through further analyses, we found that the immune infiltration level of cluster 2 was lower than that of cluster 1, and the response ability to PD1 and CLAT4 was weak. This may explain why TMB and MSI, which reflect the effect of immunotherapy, suggested that cluster 2 had a poor response to the treatment. Therefore, other methods would be required for cluster 2 patients in clinical therapy. In addition, chemotherapy was more suitable for patients in cluster 1. Cisplatin, adriamycin, etoposide, and paclitaxel could be used for patients in cluster 1, as these drugs have shown higher sensitivity there and the therapeutic effect may be better.

Through LASSO regression of 28 mRLs, 10 mRLs with independent prediction values were screened and applied to build a prognostic model. There were statistical distinctions in the 10 mRL-expressed levels between normal and tumor tissues. The outcomes of qRT-PCR were similar to the trends found in the TCGA dataset, which confirmed the prediction ability of the MRLM to a certain degree. The significant difference in the protein expression levels of m1A that exists between normal tissues and UCEC tissues once again illustrates the discrimination ability of the model, while the survival probability corresponding to high expression and low expression was also different (except AC011466.1). Beyond the testing set, training set, or entire set, the OS of patients at low risk was better than that of the high-risk group, which meant this MRLM had the potential to predict the prognosis of UCEC patients. With different clinical characteristics, the survival rate of patients in the high-risk group was also lower than that of patients in the low-risk group. Univariate and multivariate Cox regression analyses indicated that risk score was an important independent prediction factor for UCEC. The AUC value of our model ranged from 0.66 to 0.86, indicating that the prediction accuracy of the presented model was acceptable. After combining clinical features, the prediction was more accurate, suggesting that, in clinical practice, combining this model with clinical features can predict the prognosis of UCEC patients. The nomogram also suggested that MRLM had better accuracy, and the prediction ability combined with clinical factors would be superior.

GSEA indicated that low-risk samples were enriched with immune-related pathways, while high-risk samples were enriched with some tumor-related pathway. Immune cell infiltration expression in UCEC was related to clinical prognosis. In the high-risk group, the expressions of CD8 T cells, regulatory T cells (Tregs), gamma delta T cells, and activated NK cells were low, and the expressions of M1 macrophages and M2 macrophages were high. After that, the analysis of the relationship between MLRM score and immune infiltrating cells also indicated consistent results. On the one hand, MLRM could distinguish different characteristics of immune cells. The tumors of high-risk patients had less T cell infiltration, indicating T cell failure. Poor T cell infiltration in tumors was associated with immune escape [63]. Decreased immune infiltration and T cell failure met the definition of the “immune desert” phenotype [64]. This phenotype reflects a lacking of antitumor immunity and less response to ICI therapy [65]. In other words, the immune monitoring function of patients with high-risk score and cluster 2 was weakened, which is conducive to immune escape, meaning the effect of immunotherapy would be poor. Several studies have indicated that TME, which tumor cell growth and survival depend on, has had a crucial effect on tumor development. The ESTIMATE scores, immune cells, and immune function scores of low-risk patients were high, which meant better immune status and better prognosis. CSCs (cancer stem cells) take part in tumor progression, treatment resistance, and recurrence [50]. The low degree of cell differentiation and the strong characteristics of stem cells indicate that the disease may be more serious. In the future, we can try to explore therapeutic targets with stem cell characteristics.

Evaluating the response of ICI in terms of the characteristics of TME cell infiltration was a crucial step in improving the reaction rate of ICI treatment and developing new immunotherapy methods [66]. For the most part, the expressed level of immune checkpoints in the low-risk group was high, indicating that immunosuppressants acting on immune checkpoints, such as PD1, can be employed to carry out immunotherapy on low-risk groups. The above results indicated that immunosuppressants acting on immune checkpoints, such as PD1, could also be used for immunotherapy in patients who were low risk. In addition, such patients had higher IPS, indicating higher immunogenicity. The above results once again showed that the UCEC of patients at high risk belonged to cold tumor and might have a poor response to immunotherapy.

High TMB indicates a great curative effect of the PD-1/PD-L1 blockade in tumors [67]. The TMB of the low-risk group was higher than that of the high-risk group, and better curative effects could be obtained through immunotherapy. Not surprisingly, the results showed that the OS of UCEC patients with L-TMB was low, similar to the conclusion of previous studies [68]. MSI is another tumor marker reflecting the therapeutic effect of ICIs. In comparison with MSS/MSI-L samples, MSI-H samples had more immune cell infiltration and higher immunogenicity, significantly benefiting from ICI treatment [69]. MSI in low-risk groups had poor stability and a better effect on immunotherapy. In 2020, the National Comprehensive Cancer Network clinical practice guidelines in oncology pointed out that pembrolizumab was recommended for the second-line treatment of advanced endometrial cancer with MSI-H/dMMR [70]. CNV is an important source of human genetic diversity that is closely associated with many diseases through various molecular mechanisms [71]. It often appears in many RNA regulatory genes (such as genes related to m6A, m5C, m1A, m3C, and m7G) [72]. Previous studies found that CNV in m6A regulatory genes had a significant negative impact on patient survival [73]. Our genomic analysis indicated that patients in the low-risk group had more gene mutations and CNV load, which was the immune activation group.

In addition to the application of ICIS immunotherapy, chemotherapeutic drugs are also commonly used in the treatment of tumors. Cisplatin, doxorubicin, etoposide, and paclitaxel are four common chemotherapeutic drugs that can be utilised to treat many types of cancer [74–77]. Although there is no study verifying that these drugs can be applied to treat UCEC, this study has shown that low-risk groups had high sensitivity to cisplatin, doxorubicin, and etoposide, meaning chemotherapy for low-risk people may obtain better results. Recently, a study found that plumbagin played an anti-UCEC role through anti-inflammatory, immune regulation, and regulation of some crucial pathways related to anti-inflammatory and immune regulation [78]. CMap accurately screened drugs that have had specific effects on cancer stem cells, which might eventually help the clinical practice of UCEC treatment. However, up to now, there has been no report on the application of these drugs in UCEC.

Our study identified 10 mRLs to establish a prognostic signature: AC078883.1, AC027319.1, BOLA3–AS1, AC093227.1, HM13–IT1, AL645568.1, HMGN3–AS1, AC006329.1, AP003096.1, and AC011466.1. There are few articles reporting on the effect of these lncRNAs in UCEC and other tumors. Researchers once constructed a signature that had the prognosis ability of UCEC in light of five glycolysis-related lncRNAs, including BOLA3-AS1 [37]. BOLA3-AS1 could also participate in the construction of models to predict the prognosis of gastric cancer, as well as left-sided and right-sided colon cancers [79, 80]. AC006329.1 has great potential for assessing risk and supplying personal treatment for colon cancer patients [81].

At present, there is no unified prognostic biomarker for UCEC. UCEC patients at the same clinicopathological stage may have different prognoses, and it is inaccurate to judge the prognosis only by clinical characteristics. Therefore, potential and more effective biomarkers for prediction and treatment should be explored. According to the above results, we conclude that the prognosis signature may offer a reliable immune biomarker for UCEC.

Nevertheless, there are also several limitations to this study. Our research has only utilised a public database, and more prospective real data should be included to verify the clinical practicability of this signature. In addition, except for in vitro experiments, more in vivo experiments should be conducted to comprehensively explore the regulatory mechanism of these lncRNAs. In the future, we will continue to collect clinical samples and expand the sample size.

## 5. Conclusion

MRLM is an accurate and reliable biomarker for predicting the prognosis of UCEC. In this study, we constructed a prognostic model of UCEC based on mRLs. It can be used to identify potential UCEC patients at an early stage, judge the prognosis of patients, and select more effective immunotherapy or chemotherapy for patients, helping to realise individualised and accurate treatments. These results also promote the future study of the modification process and mechanism of mRLs.

## Figures and Tables

**Table 1:** Univariate and multivariate Cox regression analyses of the prognosis-related factors.

Variable	Univariable model				Multivariable model			
	HR	HR.95L	HR.95H	*P* value	HR	HR.95L	HR.95H	*P* value
Training set								
Age	2.2793	1.1469	4.5295	0.0187	1.4265	0.6649	3.0608	0.3618
Histological type	3.8830	2.1109	7.1428	0.0000	1.8967	0.9135	3.9380	0.0859
Grade	1.8751	0.7365	4.7743	0.1874				
Stage	3.2663	1.7902	5.9595	0.0001	2.3633	1.2505	4.4661	0.0081
Risk score	1.3711	1.2493	1.5047	0.0000	1.2498	1.1156	1.4001	0.0001
Testing set								
Age	1.4056	0.7379	2.6775	0.3004				
Histological type	2.4898	1.3938	4.4478	0.0021	1.0610	0.5520	2.0393	0.8591
Grade	10.5898	1.4590	76.8649	0.0196	5.2309	0.6895	39.6869	0.1095
Stage	5.2467	2.8787	9.5624	0.0000	3.8063	1.9836	7.3039	0.0001
Risk score	1.1496	1.0695	1.2357	0.0002	1.0850	1.0042	1.1722	0.0388
Entire set								
Age	1.7782	1.1121	2.8432	0.0162	1.5813	0.9755	2.5636	0.0630
Histological type	3.0435	2.0032	4.6242	0.0000	1.4812	0.9172	2.3921	0.1081
Grade	3.3631	1.4671	7.7097	0.0042	1.5113	0.6245	3.6574	0.3597
Stage	4.1162	2.7000	6.2754	0.0000	3.0580	1.9229	4.8634	0.0000
Risk score	1.2032	1.1424	1.2671	0.0000	1.1235	1.0563	1.1949	0.0002

## Data Availability

The data used to support the findings of this study are available from the corresponding author on reasonable request.

## Abbreviations

UCEC: Uterine corpus endometrial carcinoma
m1A: N1-methyladenosine
lncRNAs: Long noncoding RNAs
TMB: Tumor mutation burden
MSI: Microsatellite instability
LASSO: Least absolute shrinkage and selection operator
mRLs: m1A-related lncRNAs
TCGA: The Cancer Genome Atlas
OS: Overall survival
mRGs: m1A-related genes
PCA: Principal component analysis
AUC: Area under the curve
ROC: Receiver operating characteristic
GSEA: Gene set enrichment analysis
KEGG: Kyoto Encyclopedia of Genes and Genomes
TME: Tumor microenvironment
ssGSEA: Single-sample gene set enrichment analysis
CSCs: Cancer stem cells
mRNAsi: mRNA expression-based stemness index
IPS: Immunophenoscore
TCIA: The Cancer Imaging Archive
ICIs: Immune checkpoint inhibitors
CNV: Copy number variation
CMap: Connectivity map
MoA: Mechanism of action
Tregs: Regulatory T cells
MRLM: The model based on m1A-related lncRNA
qRT-PCR: Quantitative real-time reverse transcription-polymerase chain reaction.

## Supplementary Materials

Supplementary 1 Figure S1: flow chart and correlations between mRGs and mRLs. (A) The flow chart of our study. (B) The network of 10 mRGs and 621 mRLs. (C) Sankey relational diagram for 10 mRGs and mRLs. (D) Heat map for the correlations between 10 mRGs and the 10 prognostic mRLs.

Supplementary 2 Figure S2: consensus clustering analysis. (A) Uniform clustering cumulative distribution function (CDF), *k* = 2‐9 (*k* represents the number of clusters). (B) The change of area under CDF curve with *k* = 2‐9. (C) TCGA UCEC cohort was divided into two clusters when *k* = 2. (D) Tracking plot of the cluster when *k* = 2.

Supplementary 3 Figure S3: LASSO regression analysis. The tuning parameters of 10 mRLs were selected to cross-verify the error curve. According to the minimal criterion and 1-se criterion, perpendicular imaginary lines were drawn at the optimal value. (B) The LASSO coefficient profile of 10 mRLs and the perpendicular imaginary line was drawn at the value chosen by 10-fold cross-validation.

Supplementary 4 Figure S4: expression levels of the mRLs between normal tissues and tumor tissue evaluated by using qRT-PCR.

Supplementary 5 Figure S5: protein expression of the mRGs between normal tissues and UCEC tissue.

Supplementary 6 Figure S6: principal component analysis (PCA). The result of PCA in training set (A), testing set (B), and entire set (C).

Supplementary 7 Figure S7: effects of different clinical features and risk scores on UCEC prognosis. (A) The different expression of 10 mRLs and their clinical features between the two risk groups were shown by heat map. (B) Differences in age, stage, histological type, and grade between the two risk groups. (C) The difference of the risk scores of patients with different clinical characteristics. (D) Comparison of survival probability between high- and low-risk groups under different clinical characteristic. (E) The difference of the risk scores of patients with different expression levels of mRLs.

Supplementary 8 Figure S8: mutation of somatic and genes in three sets. Somatic mutation count of high- and low-risk groups in the training set (A), testing set (B), and entire set (C). The mutation proportion of PTEN (D–F), ARID1A (G–I), PIK3CA (J–L), POLE (M–O), TP53 (P–R), and TTN (S–U) in the two groups.

Supplementary 9 Table S1: the clinical characteristics of UCEC patients in the TCGA database.

Supplementary 10 Table S2: primers used in PCR application.

Supplementary 11 Table S3: univariate Cox regression analysis of mRLs.

Supplementary 12 Table S4: LASSO regression analysis of mRLs in training set.

Supplementary 13 Table S5: output from the connectivity map.
