# Supplementary figures and images for: N1-Methyladenosine-Related lncRNAs Are Potential Biomarkers for Predicting Prognosis and Immune Response in Uterine Corpus Endometrial Carcinoma
Source: Oxid Med Cell Longev. 2022 Jul 31;2022:2754836. doi: 10.1155/2022/2754836 (PMC9372539; doi:10.1155/2022/2754836)

A

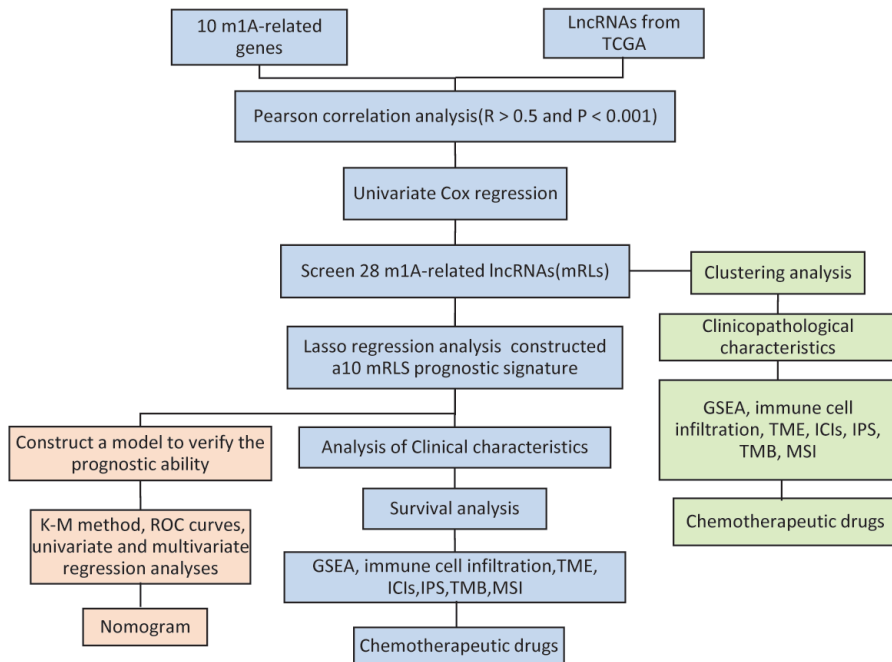

B

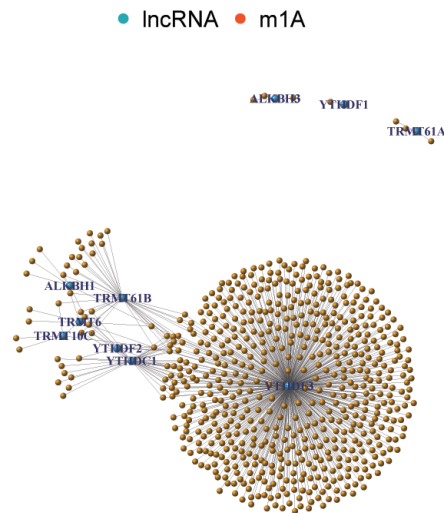

C

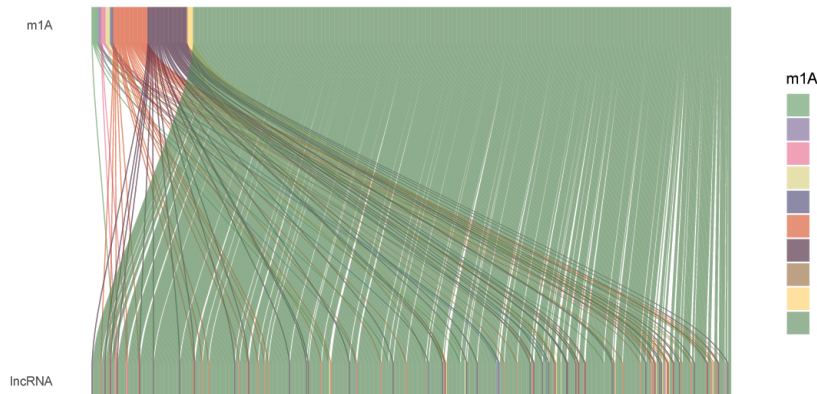

D

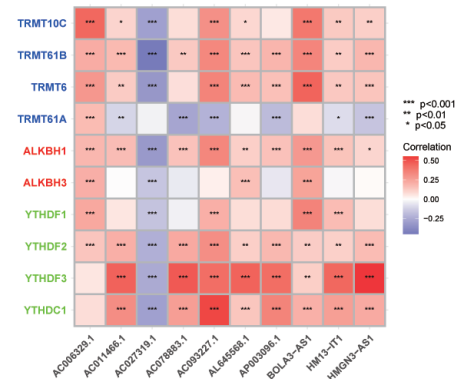

Supplement: Supplementary 1 — Figure S1: flow chart and correlations between mRGs and mRLs. (A) The flow chart of our study. (B) The network of 10 mRGs and 621 mRLs. (C) Sankey relational diagram for 10 mRGs and mRLs. (D) Heat map for the correlations between 10 mRGs and the 10 prognostic mRLs. [file 2754836.f1.pdf]

A

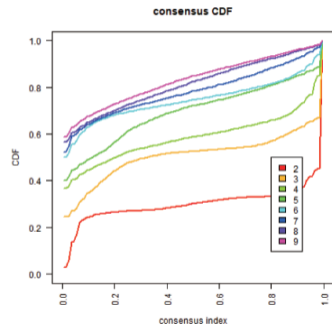

B

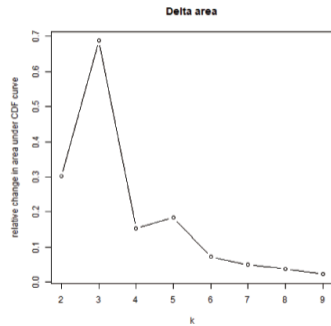

C

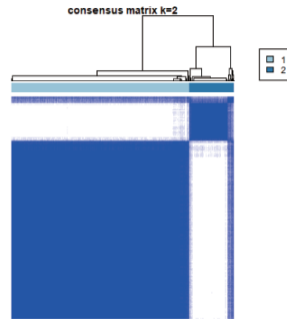

D

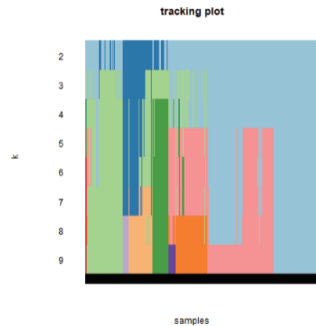

Supplement: Supplementary 2 — Figure S2: consensus clustering analysis. (A) Uniform clustering cumulative distribution function (CDF), k = 2‐9 (k represents the number of clusters). (B) The change of area under CDF curve with k = 2‐9. (C) TCGA UCEC cohort was divided into two clusters when k = 2. (D) Tracking plot of the cluster when k = 2. [file 2754836.f2.pdf]

A

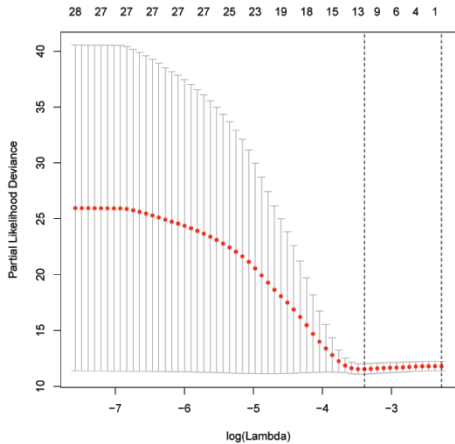

B

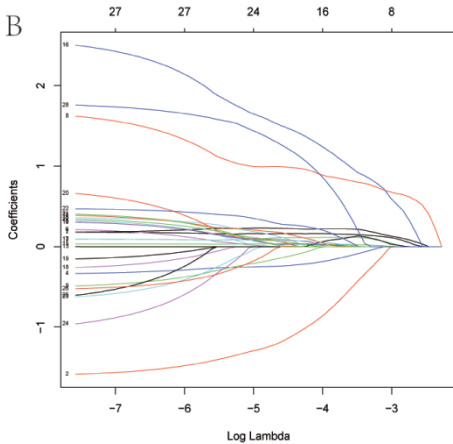

Supplement: Supplementary 3 — Figure S3: LASSO regression analysis. The tuning parameters of 10 mRLs were selected to cross-verify the error curve. According to the minimal criterion and 1-se criterion, perpendicular imaginary lines were drawn at the optimal value. (B) The LASSO coefficient profile of 10 mRLs and the perpendicular imaginary line was drawn at the value chosen by 10-fold cross-validation. [file 2754836.f3.pdf]

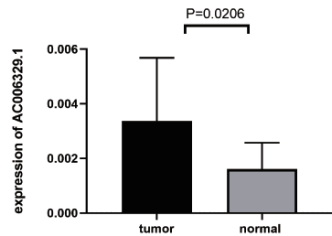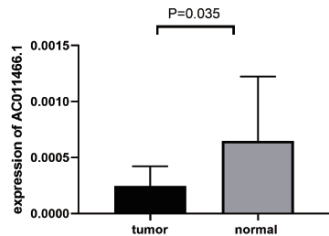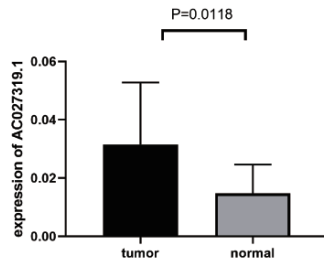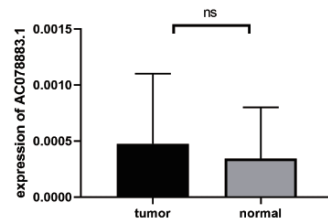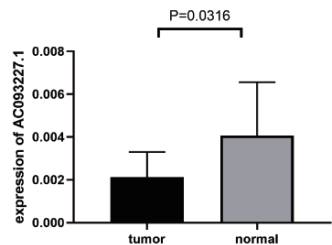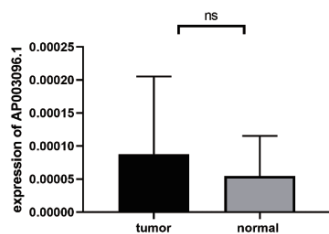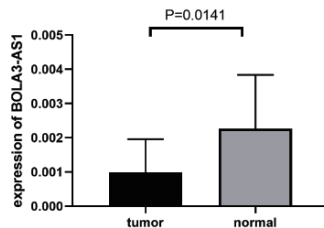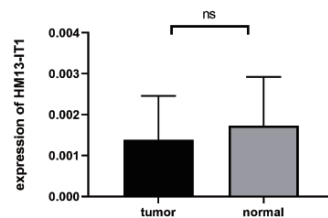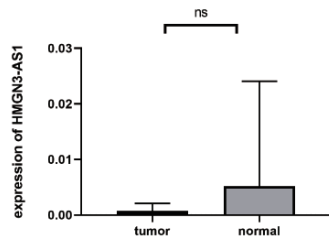

Supplement: Supplementary 4 — Figure S4: expression levels of the mRLs between normal tissues and tumor tissue evaluated by using qRT-PCR. [file 2754836.f4.pdf]

A

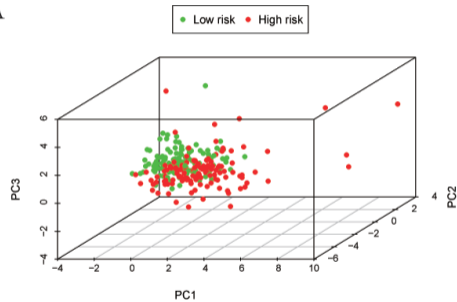

B

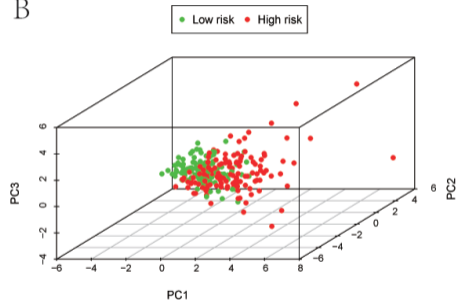

C

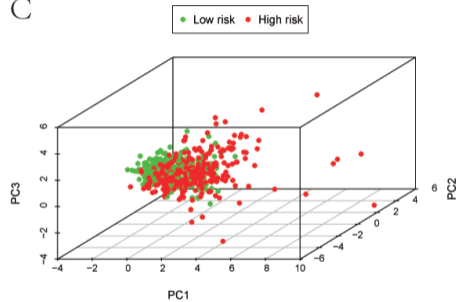

Supplement: Supplementary 6 — Figure S6: principal component analysis (PCA). The result of PCA in training set (A), testing set (B), and entire set (C). [file 2754836.f6.pdf]

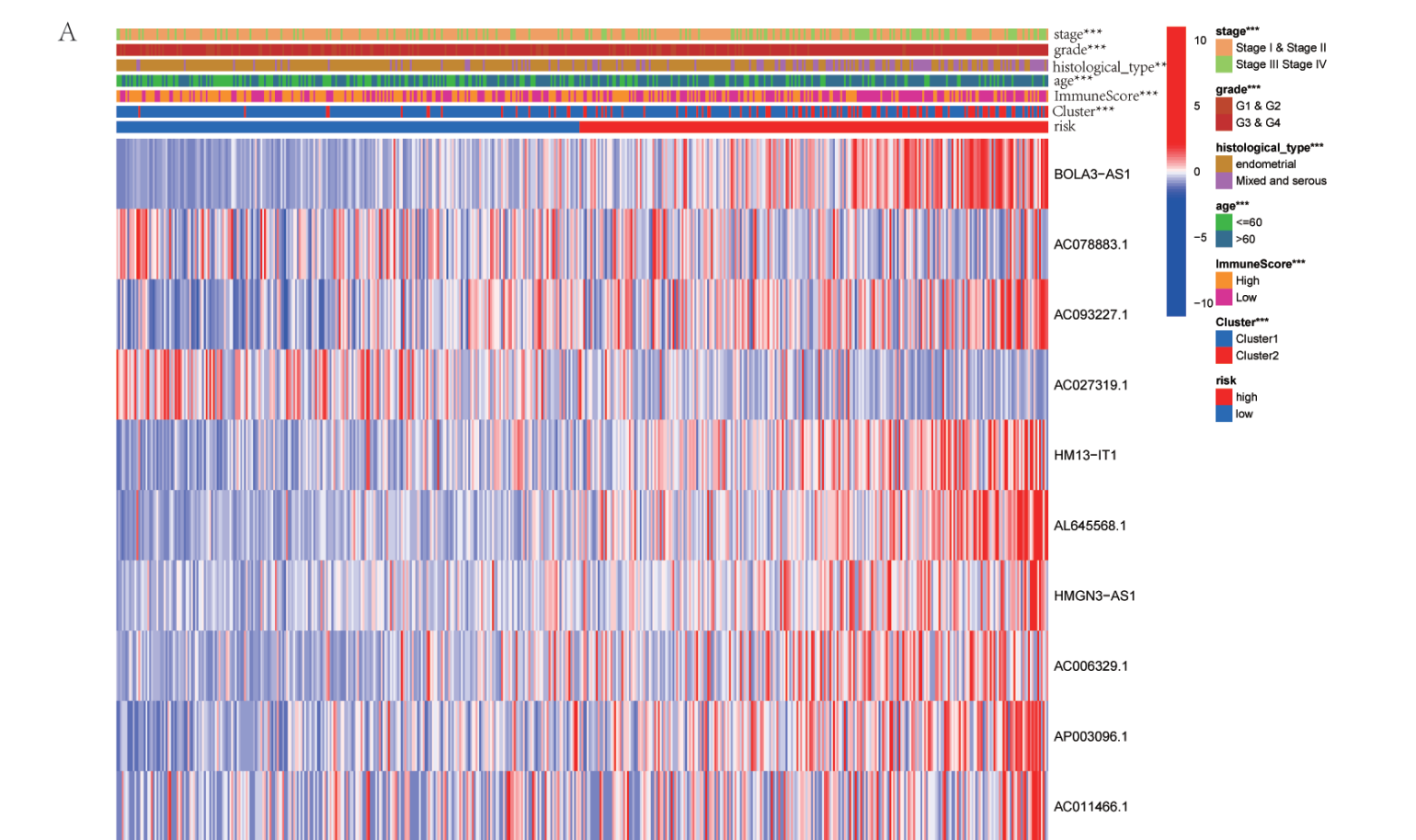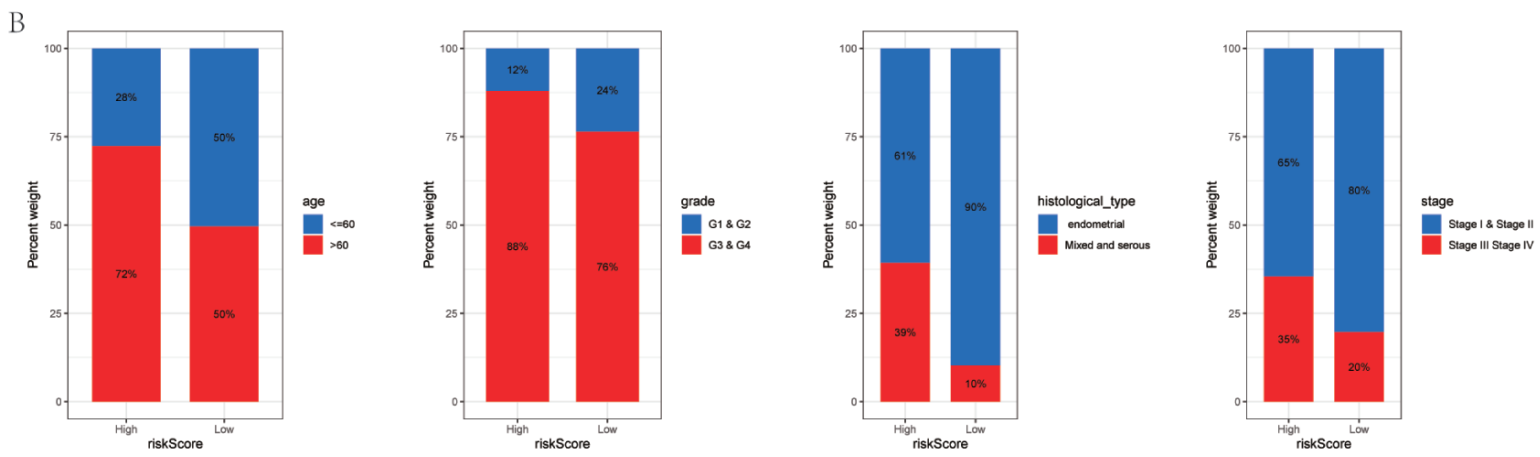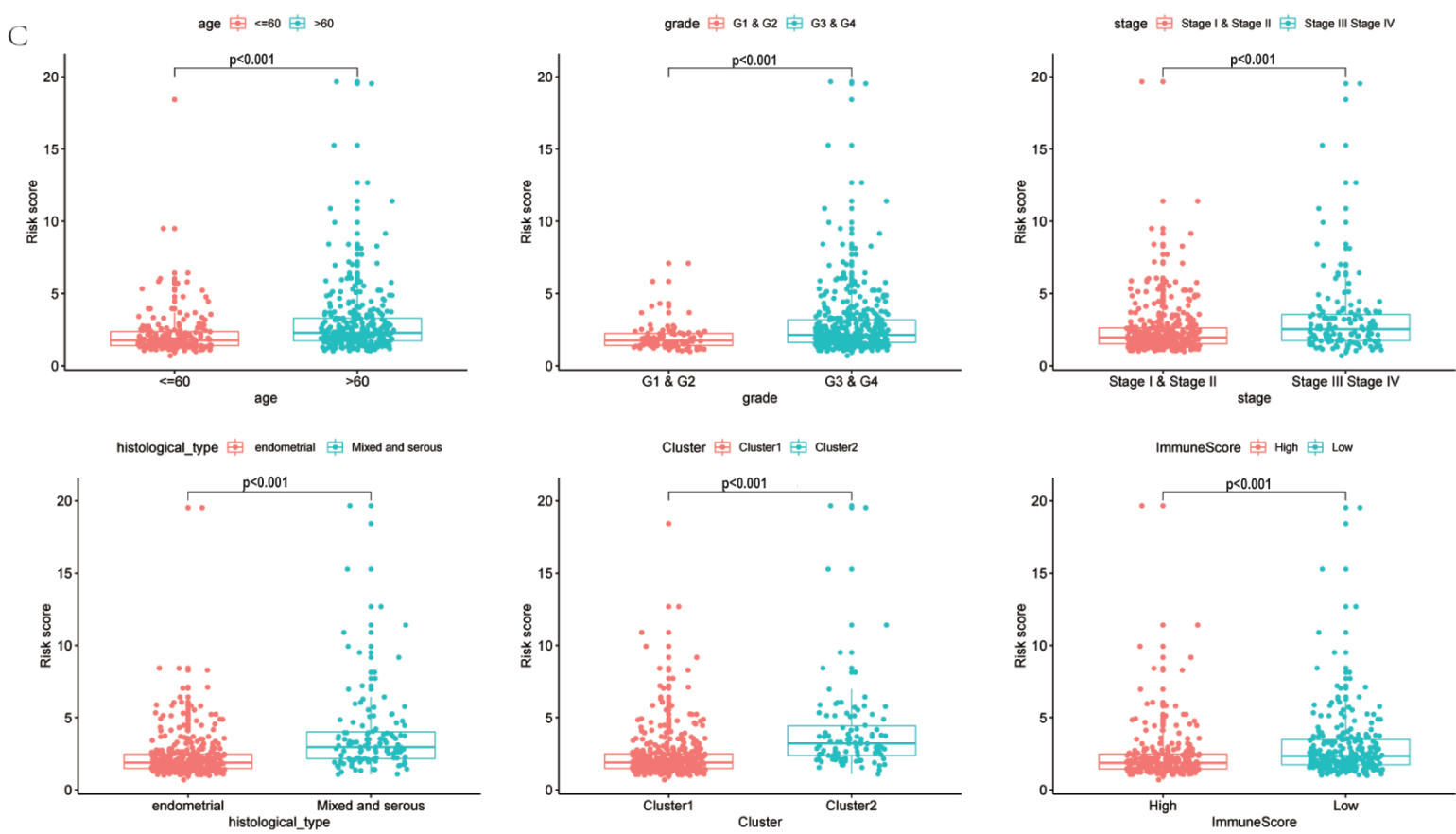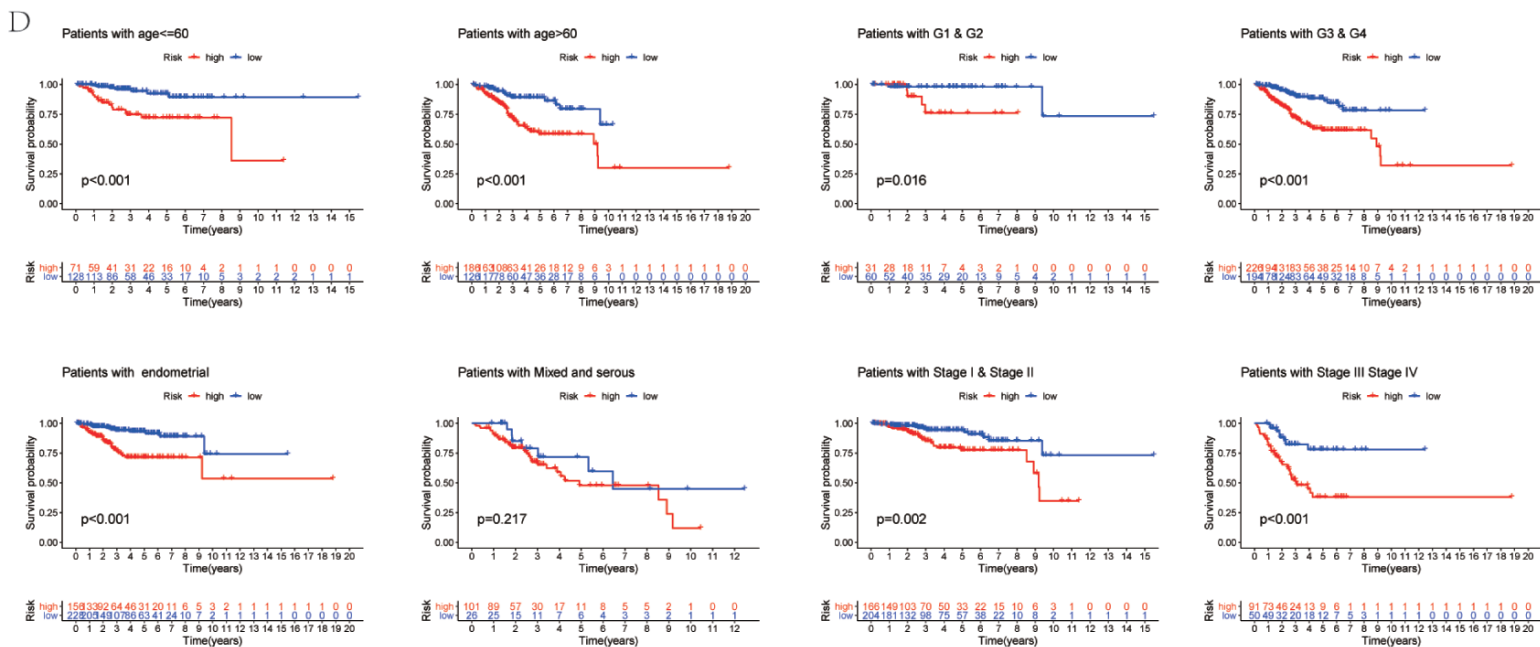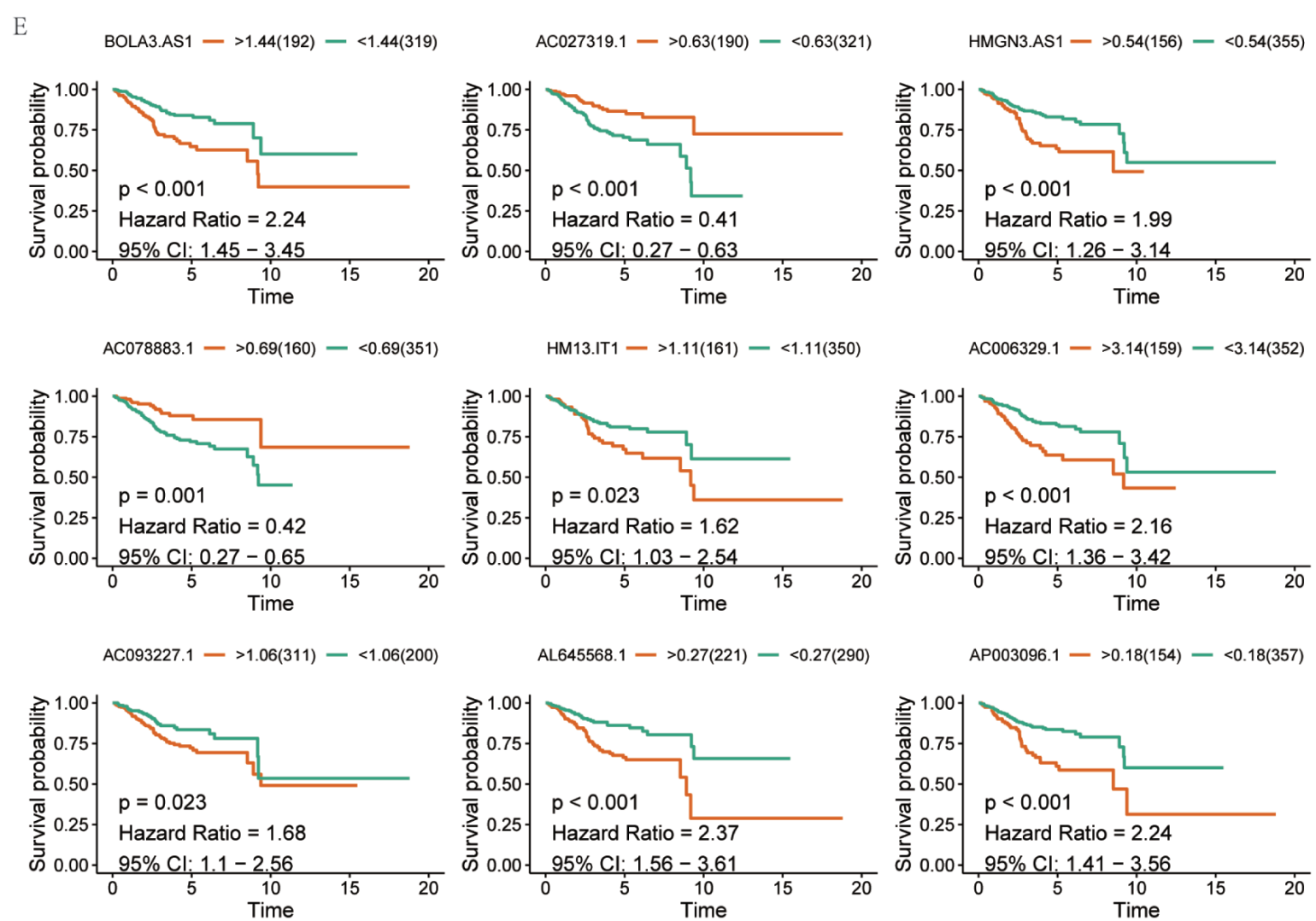

Supplement: Supplementary 7 — Figure S7: effects of different clinical features and risk scores on UCEC prognosis. (A) The different expression of 10 mRLs and their clinical features between the two risk groups were shown by heat map. (B) Differences in age, stage, histological type, and grade between the two risk groups. (C) The difference of the risk scores of patients with different clinical characteristics. (D) Comparison of survival probability between high- and low-risk groups under different clinical characteristic. (E) The difference of the risk scores of patients with different expression levels of mRLs. [file 2754836.f7.pdf]

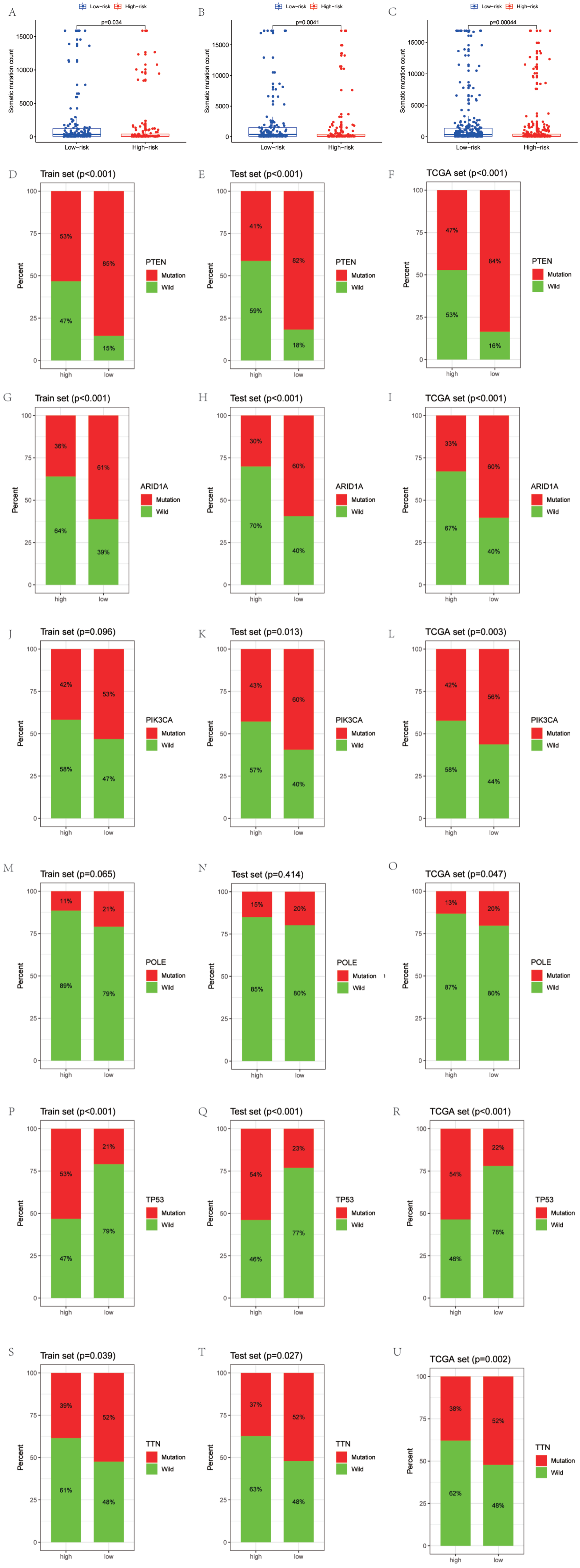

Supplement: Supplementary 8 — Figure S8: mutation of somatic and genes in three sets. Somatic mutation count of high- and low-risk groups in the training set (A), testing set (B), and entire set (C). The mutation proportion of PTEN (D–F), ARID1A (G–I), PIK3CA (J–L), POLE (M–O), TP53 (P–R), and TTN (S–U) in the two groups. [file 2754836.f8.pdf]
