# Supplementary material for: N1-Methyladenosine-Related lncRNAs Are Potential Biomarkers for Predicting Prognosis and Immune Response in Uterine Corpus Endometrial Carcinoma
Source: Oxid Med Cell Longev. 2022 Jul 31;2022:2754836. doi: 10.1155/2022/2754836 (PMC9372539; doi:10.1155/2022/2754836)
Supplement: Supplementary 5 — Figure S5: protein expression of the mRGs between normal tissues and UCEC tissue. [file 2754836.f5.pdf]

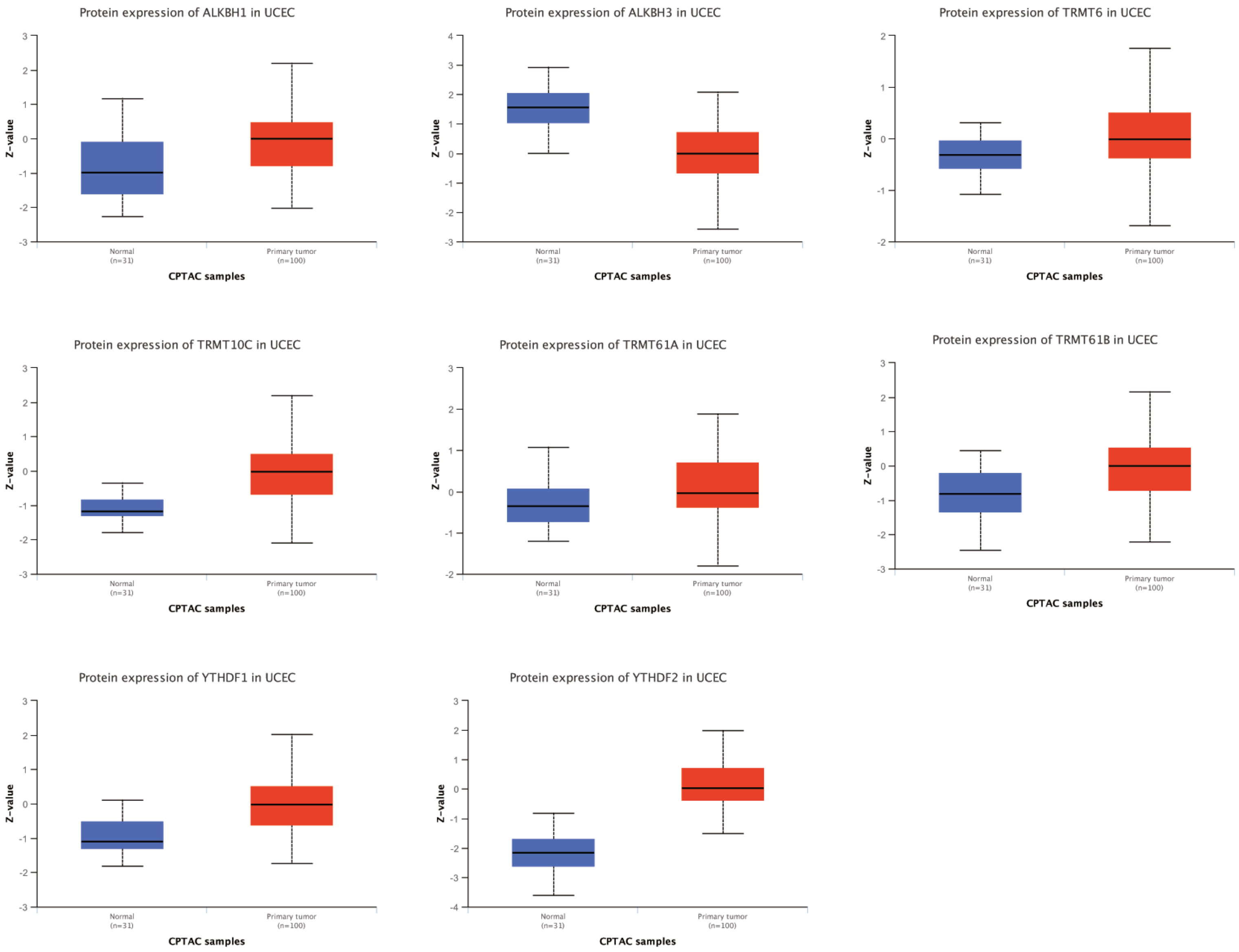
Protein expression of ALKBH1 in UCEC
Z-value
3 2 1 0 -1 -2 -3
Normal (n=31)
Primary tumor (n=100)
CPTAC samples
Protein expression of ALKBH3 in UCEC
Z-value
4 3 2 1 0 -1 -2 -3
Normal (n=31)
Primary tumor (n=100)
CPTAC samples
Protein expression of TRMT6 in UCEC
Z-value
2 1 0 -1 -2
Normal (n=31)
Primary tumor (n=100)
CPTAC samples
Protein expression of TRMT10C in UCEC
Z-value
3 2 1 0 -1 -2 -3
Normal (n=31)
Primary tumor (n=100)
CPTAC samples
Protein expression of TRMT61A in UCEC
Z-value
3 2 1 0 -1 -2
Normal (n=31)
Primary tumor (n=100)
CPTAC samples
Protein expression of TRMT61B in UCEC
Z-value
3 2 1 0 -1 -2 -3
Normal (n=31)
Primary tumor (n=100)
CPTAC samples
Protein expression of YTHDF1 in UCEC
Z-value
3 2 1 0 -1 -2 -3
Normal (n=31)
Primary tumor (n=100)
CPTAC samples
Protein expression of YTHDF2 in UCEC
Z-value
3 2 1 0 -1 -2 -3 -4
Normal (n=31)
Primary tumor (n=100)
CPTAC samples
