# Supplementary material for: N1-Methyladenosine-Related lncRNAs Are Potential Biomarkers for Predicting Prognosis and Immune Response in Uterine Corpus Endometrial Carcinoma
Source: Oxid Med Cell Longev. 2022 Jul 31;2022:2754836. doi: 10.1155/2022/2754836 (PMC9372539; doi:10.1155/2022/2754836)
Supplement: Supplementary 9 — Table S1: the clinical characteristics of UCEC patients in the TCGA database. [file 2754836.f9.pdf]

Table S1 The clinical characteristics of UCEC patients in the TCGA database.

Covariates	Type	Total	Test	Train
age	＜=60	199(38.94%)	92(36.08%)	107(41.8%)
age	＞60	312(61.06%)	163(63.92%)	149(58.2%)
histological_type	endometrial	384(75.15%)	181(70.98%)	203(79.3%)
histological_type	Mixed and serous	127(24.85%)	74(29.02%)	53(20.7%)
grade	G1 & G2	91(17.81%)	48(18.82%)	43(16.8%)
grade	G3 & G4	420(82.19%)	207(81.18%)	213(83.2%)
stage	Stage I & Stage II	370(72.41%)	183(71.76%)	187(73.05%)
stage	Stage III Stage IV	141(27.59%)	72(28.24%)	69(26.95%)
