# Supplementary material for: N1-Methyladenosine-Related lncRNAs Are Potential Biomarkers for Predicting Prognosis and Immune Response in Uterine Corpus Endometrial Carcinoma
Source: Oxid Med Cell Longev. 2022 Jul 31;2022:2754836. doi: 10.1155/2022/2754836 (PMC9372539; doi:10.1155/2022/2754836)
Supplement: Supplementary 10 — Table S2: primers used in PCR application. [file 2754836.f10.pdf]

Table S2 Primers used in PCR application

Gene	Forward primers	Reverse primer
BOLA3-AS1	AGTCAGAAGCTCCGAGGCTA	TTTGCGGACAGTTCTACCCC
AC078883.1	ATCTCAAGTGAACAGGCCAAGAA	GTGTGGAATGCCTCGTTCCT
AC093227.1	ACAGCTACTCTCCAGGAAAAGG	TGCTCATTTTCTCAACCTGCTG
AC027319.1	TGGCCTGTATGCAGTTGTGG	TGTTTACACCGATGTCCCTGA
HM13-IT1	CCCAAGGCCTGTGTTACTCC	CCTCATGTCCTGGATCCCCT
HMGN3-AS1	CCCCGAACTGCGGCCTA	CTTTCTCTCCAGTGACGCGG
AP003096.1	TTCTTGCCTGGCATTCCGTT	CCACCACCTGTGTCGTAGAAG
AC011466.1	CCACGCTGTTTTGAAGAGCC	GAAGGGCGTGAGGACATCAT
GAPDH	GCACCGTCAAGGCTGAGAAC	GGATCTCGCTCCTGGAAGATG
