# Supplementary material for: N1-Methyladenosine-Related lncRNAs Are Potential Biomarkers for Predicting Prognosis and Immune Response in Uterine Corpus Endometrial Carcinoma
Source: Oxid Med Cell Longev. 2022 Jul 31;2022:2754836. doi: 10.1155/2022/2754836 (PMC9372539; doi:10.1155/2022/2754836)
Supplement: Supplementary 11 — Table S3: univariate Cox regression analysis of mRLs. [file 2754836.f11.pdf]

Table S3 Univariate Cox regression analysis of mRLs

gene	HR	HR.95L	HR.95H	p-value
BOLA3-AS1	1.3048	1.1568	1.4717	0.0000
AC078883.1	0.3806	0.1852	0.7820	0.0086
AC093227.1	1.3556	1.1069	1.6603	0.0033
AC027319.1	0.4268	0.2561	0.7114	0.0011
AL078644.1	2.8777	1.4146	5.8541	0.0035
AL049539.1	1.4328	1.1414	1.7988	0.0019
HM13-IT1	1.4375	1.1987	1.7238	0.0001
AL645568.1	3.3838	1.8640	6.1428	0.0001
NBAT1	1.3232	1.0718	1.6336	0.0092
FMR1-IT1	1.4578	1.1154	1.9054	0.0058
LRRC8C-DT	3.6737	1.4920	9.0455	0.0047
AL133243.2	1.2408	1.0780	1.4282	0.0026
HMGN3-AS1	1.4796	1.1078	1.9763	0.0080
TPM1-AS	2.1302	1.3048	3.4778	0.0025
AC006329.1	1.0986	1.0397	1.1608	0.0008
AP003096.1	8.2753	2.5536	26.8179	0.0004
NNT-AS1	1.2313	1.0730	1.4129	0.0030
AC074117.1	1.3876	1.1421	1.6858	0.0010
SOS1-IT1	1.2903	1.1360	1.4657	0.0001
LINC01126	2.5007	1.2655	4.9414	0.0083
AL606763.1	1.9727	1.2693	3.0659	0.0025
AC092953.2	2.2176	1.2689	3.8758	0.0052
AL031667.3	1.7529	1.1444	2.6849	0.0099
AL035530.2	4.4538	1.8565	10.6848	0.0008
AC114947.2	2.8938	1.3980	5.9898	0.0042
AC244517.7	1.8161	1.2804	2.5758	0.0008
AC244517.1	1.4300	1.1339	1.8034	0.0025
AC011466.1	16.8753	3.4921	81.5499	0.0004
