# Supplementary material for: N1-Methyladenosine-Related lncRNAs Are Potential Biomarkers for Predicting Prognosis and Immune Response in Uterine Corpus Endometrial Carcinoma
Source: Oxid Med Cell Longev. 2022 Jul 31;2022:2754836. doi: 10.1155/2022/2754836 (PMC9372539; doi:10.1155/2022/2754836)
Supplement: Supplementary 12 — Table S4: LASSO regression analysis of mRLs in training set. [file 2754836.f12.pdf]

Table S4 Lasso analysis of mRLs in training set.

Gene	Coef
BOLA3-AS1	0.1426
AC078883.1	-0.3418
AC093227.1	0.1069
AC027319.1	-0.0669
HM13-IT1	0.2003
AL645568.1	0.7864
HMGN3-AS1	0.1220
AC006329.1	0.0208
AP003096.1	0.8807
AC011466.1	0.0403
