# Supplementary material for: N1-Methyladenosine-Related lncRNAs Are Potential Biomarkers for Predicting Prognosis and Immune Response in Uterine Corpus Endometrial Carcinoma
Source: Oxid Med Cell Longev. 2022 Jul 31;2022:2754836. doi: 10.1155/2022/2754836 (PMC9372539; doi:10.1155/2022/2754836)
Supplement: Supplementary 13 — Table S5: output from the connectivity map. [file 2754836.f13.pdf]

Table S5 Output from the Connectivity Map

rank	CMap name	mean	n	enrichment	*P*	specificity	percent non-null
1	iloprost	-0.354	3	-0.902	0.00178	0	66
2	dirithromycin	0.466	3	0.889	0.00262	0	66
3	acebutolol	-0.337	5	-0.718	0.00391	0	60
4	colistin	-0.265	4	-0.776	0.00513	0.0231	50
5	cefamandole	-0.335	4	-0.762	0.00656	0.0083	50
6	W-13	-0.531	2	-0.943	0.00716	0	100
7	papaverine	0.388	4	0.744	0.00804	0.0196	50
8	Trolox C	0.364	4	0.683	0.0217	0.0081	50
9	mesoridazine	0.157	4	0.683	0.02174	0	50
10	rifabutin	0.48	3	0.775	0.02299	0.1563	66
11	harpagoside	-0.313	4	-0.680	0.02325	0.0637	50
12	valinomycin	-0.251	4	-0.646	0.03756	0.1429	50
13	metergoline	0.161	4	0.645	0.03823	0.1675	50
14	piroxicam	0.352	4	0.631	0.04595	0.0426	50
15	naltrexone	0.142	5	0.558	0.05443	0.1791	60
16	isotretinoin	-0.356	4	-0.615	0.0555	0.0635	50
17	strophanthidin	-0.279	4	-0.613	0.05662	0.0674	50
18	cromoglicic acid	-0.253	2	-0.820	0.06463	0.0553	50
19	5255229	0.442	2	0.821	0.06487	0.0526	50
20	kaempferol	-0.311	4	-0.601	0.06618	0.0833	50
